# Landscape of Genetic Mutations in Appendiceal Cancers

**DOI:** 10.3390/cancers15143591

**Published:** 2023-07-12

**Authors:** Marian Constantin, Cristina Mătanie, Livia Petrescu, Alexandra Bolocan, Octavian Andronic, Coralia Bleotu, Mihaela Magdalena Mitache, Sorin Tudorache, Corneliu Ovidiu Vrancianu

**Affiliations:** 1 Institute of Biology of Romanian Academy, 060031 Bucharest, Romania; cvgmarian@gmail.com; 2 The Research Institute of the University of Bucharest (ICUB), 050095 Bucharest, Romania; ovidiu.vrancianu@yahoo.com; 3 Department of Anatomy, Animal Physiology and Biophysics (DAFAB), Faculty of Biology, University of Bucharest, 050095 Bucharest, Romania; cristina.matanie@bio.unibuc.ro (C.M.); livia.petrescu@bio.unibuc.ro (L.P.); 4 Carol Davila University of Medicine and Pharmacy, 050474 Bucharest, Romania; andronicoctavian@gmail.com; 5 Life, Environmental and Earth Sciences Division, The Research Institute of the University of Bucharest (ICUB), 050095 Bucharest, Romania; cbleotu@yahoo.com; 6 Stefan S. Nicolau Institute of Virology, 030304 Bucharest, Romania; 7 Faculty of Medicine, “Titu Maiorescu” University, 040441 Bucharest, Romania; magda.mitache@yahoo.com (M.M.M.); soryntudorache@yahoo.com (S.T.); 8 Microbiology—Immunology Department, Faculty of Biology, University of Bucharest, 050095 Bucharest, Romania; 9 National Institute of Research and Development for Biological Sciences, 060031 Bucharest, Romania

**Keywords:** appendix tumor, KRAS genes, point mutations, signaling pathways, adenocarcinomas

## Abstract

**Simple Summary:**

An analysis of the presence of mutations of 105 genes in appendiceal cancers through the lens of the reviewed literature supports the view that in most of them, the inactivation of tumor suppressor genes, such as *TP53* and *SMAD4*, is required in parallel with the reactivation of genes with oncogenic potentials, such as *KRAS*, *GNAS*, and *BRAF*, which support the main tumor processes, cell proliferation, angiogenesis, and evasion of apoptosis. Of all appendiceal cancers, the most mutated genes are reported in mucinous neoplasms of the appendix, not including those in the RAS–RAF–MEK–ERK signaling pathway, followed by low-grade appendiceal mucinous neoplasms, appendiceal goblet cell adenocarcinomas, and mucinous adenocarcinomas of the appendix, in which this signaling pathway is most frequently affected, showing its importance in their tumorigenesis. Microsatellite instability rarely occurs in appendix cancers, being reported only in adenocarcinomas.

**Abstract:**

In appendiceal cancers, the most frequently mutated genes are (i) *KRAS*, which, when reactivated, restores signal transduction via the RAS–RAF–MEK–ERK signaling pathway and stimulates cell proliferation in the early stages of tumor transformation, and then angiogenesis; (ii) *TP53*, whose inactivation leads to the inhibition of programmed cell death; (iii) *GNAS*, which, when reactivated, links the cAMP pathway to the RAS–RAF–MEK–ERK signaling pathway, stimulating cell proliferation and angiogenesis; (iv) *SMAD4*, exhibiting typical tumor-suppressive activity, blocking the transmission of oncogenic TGFB signals via the SMAD2/SMAD3 heterodimer; and (v) *BRAF*, which is part of the RAS–RAF–MEK–ERK signaling pathway. Diverse mutations are reported in other genes, which are part of secondary or less critical signaling pathways for tumor progression, but which amplify the phenotypic diversity of appendiceal cancers. In this review, we will present the main genetic mutations involved in appendix tumors and their roles in cell proliferation and survival, and in tumor invasiveness, angiogenesis, and acquired resistance to anti-growth signals.

## 1. Introduction

The vermiform appendix is a cylindrical organ with an average length of 9 cm (5–35 cm) and a transverse diameter of 6 mm, attached to the cecum about 2 cm from its junction with the jejunum. Its origin is located near the iliocecal valve, in the cecal fundus or the posteromedial border of the cecum, and its apex hangs freely in the abdomen, with different orientations [1,2]: retrocecal/retrocolic, pelvic, post-ileal, subcecal, pre-ileal, and paracecal [3], in most cases adopting the retrocecal position [2]. Although considered an atavic organ, the vermiform appendix secretes about 2–3 mL of mucinous fluid each day and appears to have an immunoprotective and lymphatic function, especially in childhood, contributing to the maturation of B lymphocytes and to the production of immunoglobulin A, a function of recolonization of the colon with beneficial bacteria, which it stores, and an endocrine function, through the production and secretion of certain amines and hormones [1,2].

Throughout life, the appendix can be the site of various pathologies, categorized into inflammatory pathologies (acute appendicitis), pathologies related to congenital anomalies of the appendix and other related diseases, and tumors of the appendix. Tumors of the appendix are very rare pathologies and are classified by the World Health Organization [4,5] into Hyperplastic polyps, Sessile serrated lesion without dysplasia, Serrated dysplasia (low or high grade), Appendiceal mucinous neoplasms (low or high grade), Appendiceal adenocarcinomas NOS (mucinous and signet ring cell), Undifferentiated carcinomas NOS; Goblet cell adenocarcinoma, and Appendiceal neuroendocrine neoplasms (well-differentiated neuroendocrine tumors: neuroendocrine tumors NOS, neuroendocrine tumor, grades 1–3, L-cell tumor, glucagon-like peptide-producing tumor, PP/PYY-producing tumor, enterochromaffin-cell carcinoid, and serotonin-producing carcinoid; poorly differentiated neuroendocrine carcinomas *or* neuroendocrine carcinoma NOS, with large or small cells; and mixed neuroendocrine–non-neuroendocrine neoplasms). In these tumors, the most common mutations occur in the *KRAS* gene, in addition to which several mutations are common and considered necessary in the epidemiology of appendiceal cancers, including those that occur in *TP53*, *GNAS*, *SMAD4*, and *BRAF* genes, which occur with a frequency of more than 10% in cases of appendiceal cancer. Other mutations occur less frequently or are mentioned only in single cases. Some genes are predominantly active during the embryonic period, playing critical roles in cell proliferation and evading the apoptotic pathway, thus contributing to the growth and development of the embryo and fetus. However, after birth, they must diminish or cease their function, partially, totally, or conditionally (by various physiological processes) silenced. Their reactivation turns them into proto-oncogenes and is essential in initiating tumor processes. In cancer, genes whose expression products are involved in the uptake of extracellular biological signals and their transmission to the nucleus, activating factors that stimulate cell proliferation while inhibiting apoptotic signals are essential. In the case of tumor growth beyond the size that allows oxygenation and feeding by diffusion, it is necessary to activate signaling pathways that inhibit cell differentiation and stimulate angiogenesis, invasiveness, and tumor metastasis. After the chemotherapeutic treatment has started, cell clones resistant to the chemical agent are selected.

**Figure 1:**
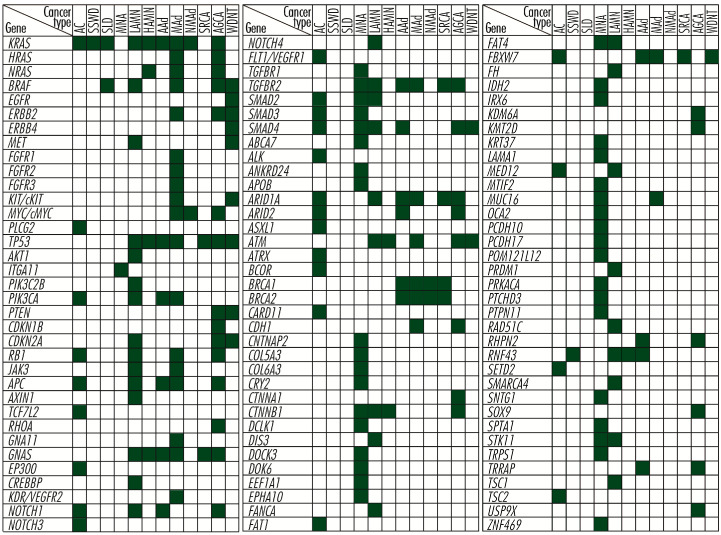
The 105 genes in which mutations are reported in appendiceal cancers. AC, appendiceal carcinoma; SSWD, sigmoid sinus wall dehiscence; SLD, single-lineage dysplasia; MNA, myelocytomatosis amplification; LAMN, low-grade appendiceal mucinous neoplasm; HAMN, high-grade appendiceal mucinous neoplasm; AAd, appendiceal adenocarcinoma; MAd, mucinous adenocarcinoma; SRCA, extragastric signet ring cell adenocarcinoma; AGCA, advanced gastric cancer; WDNT, well-differentiated neuroendocrine tumor. Green, genes reported in appendiceal cancers; white, genes not associated with appendiceal cancers.

Strictly in appendiceal cancers, most mutations tend to occur in genes whose products are involved in stimulating cell proliferation, such as the activating mutations in *RAS* and *RAF* gene families; in evading apoptosis, such as the activating mutations in *GNAS* gene and *RAS* and *RAF* gene families; as well as silencing mutations in *TP53* or *RB1* genes, or in acquiring resistance to anti-growth signals, such as the mutations in *SMAD* genes family [6,7,8,9,10,11,12,13,14,15,16]. The description of these genes is made by grouping them, generally, according to the biological processes in which their products are involved, without exhausting the range of functions they perform in the body. As is well known, some proteins perform several functions in a manner dependent on the signaling pathway through which they transmit biological signals, so placing them in one category or another is not likely to include them exclusively in these. Thus, *RAS*, *RAF*, *EGFR/ERBB*, and *FGFR* gene families, and *MET*, *KIT/cKIT*, *MYC/c-MYC*, *PLCG2*, *TP53*, *AKT1*, *ITGA11*, *PIK3C2B*, *PIK3CA*, *PTEN*, *CDKN1B*, *CDKN2A*, *RB1*, *JAK3*, *APC*, *AXIN1*, and *TCF7L2* genes are involved principally in *cell proliferation*; the *RHOA* gene, in cell survival and tumor invasiveness/metastasis; *GNA11*, *GNAS*, *EP300*, *CPB/CREBBP*, *KDR/VEGFR2*, *NOTCH1*, *NOTCH3*, *NOTCH4*, and *FLT1/VEGFR1* genes, in angiogenesis; and *SMAD2*, *SMAD3*, *SMAD4*, *SMADA*, *TGFBR1*, and *TGFBR2* genes, in acquiring insensitivity to anti-growth signals. A vast number of genes, *ABCA7*, *ALK*, *ANKRD24*, *APOB*, *ARID1A*, *ARID2*, *ASXL1*, *ATM*, *ATRX*, *BCOR*, *BRCA1*, *BRCA2*, *CARD11*, *CDH1*, *CNTNAP2*, *COL5A3*, *COL6A3*, *CRY2*, *CTNNA1*, *CTNNB1*, *DCLK1*, *DIS3*, *DOCK3*, *DOK6*, *EEF1A1*, *EPHA10*, *FANCA*, *FAT1*, *FAT4*, *FBXW7*, *FH*, *IDH2*, *IRX6*, *KDM6A*, *KMT2D*, *KRT37*, *LAMA1*, *MED12*, *MLL2*, *MTIF2*, *MUC16*, *OCA2*, *PCDH10*, *PCDH17*, *POM121L12*, *PRDM1*, *PRKACA*, *PTCHD3*, *PTPN11*, *RAD51C*, *RHPN2*, *RNF43*, *SETD2*, *SMARCA4*, *SNTG1*, *SOX9*, *SPTA1*, *STK11*, *TRPS1*, *TRRAP*, *TSC1*, *TSC2*, *USP9X*, and *ZNF4699* (Figure 1), which in appendiceal cancers occur mutated less frequently or in single cases, are involved in other processes related to tumor growth and development and are grouped separately, but without considering them less important.

In this review, we will present the genetic mutations involved in appendix tumors, including genes whose proteins are involved in cell proliferation and survival; tumor invasiveness, angiogenesis, and acquired insensitivity to anti-growth signals; and different signaling pathways which are modified in appendix cancer.

## 2. Mutations in Genes Whose Proteins Are Involved in Cell Proliferation

**Figure 2:**
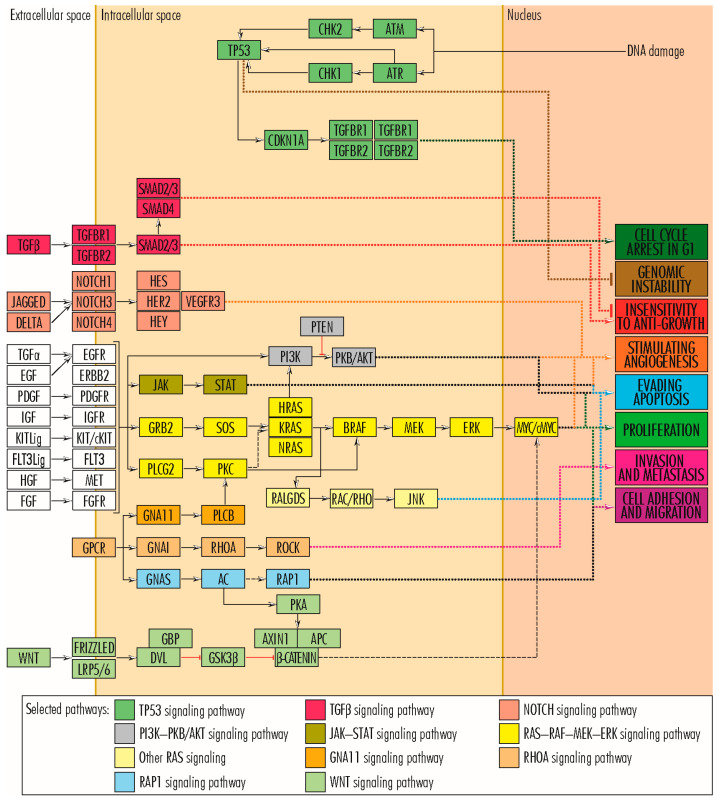
The signaling pathways important in cancer (adapted from KEGG Pathways, 2020 [14]).

Cancer cell proliferation is stimulated primarily by the RAS–RAF–MEK–ERK signaling pathway and secondarily by other pathways such as PI3K–PKB/AKT and WNT (Figure 2). In the RAS–RAF–MEK–ERK signaling pathway, which comprises the sequence of proteins EGF/TGFA–EGFR(EGF–ERBB2/PDGF–PDGFR/KITLG–KIT/FGF–FGFR/FLT3LG–FLT3/IGF–IGF1R/HGF–MET)–GRB2–SOS–RAS–RAF–MEK–ERK–c-FOS/c-JUN/c-MYC/ETS1–cyclin D1, a central role is played by members of the *RAS* gene family (*KRAS*–*Kirsten ras oncogene homolog*, *HRAS*–*HRAS proto-oncogene* and *NRAS*–*Neuroblastoma RAS Viral Oncogene Homolog*), which encodes GTPases involved in extracellular signal transduction between the cell membrane and the Golgi apparatus. They link the RAS–RAF–MEK–ERK signaling pathway to other signaling pathways, such as PI3K–PKB/AKT, involved in cell proliferation and evading cell apoptosis. In addition to RAS family proteins, the RAS–RAF–MEK–ERK signaling pathway also includes members of the RAF, EGFR, and FGFR families and the MET, KIT, MYC, and PLCG2 proteins, which can bare mutations in some appendiceal cancers (Figure 2). Physiologically, the WNT signaling pathway is an essential mechanism in regulating tissue morphogenesis and repair during embryogenesis. However, when it functions abnormally in adults, it is associated with several types of cancers, including colorectal, breast, lung, oral, cervical, and hematopoietic neoplasms [17]. Abnormalities of the WNT signaling pathway may include mutations in APC, AXIN1, RHOA, and TCF7L2 genes, reported in some appendiceal cancers. On the other hand, the TP53 protein plays a significant role in ensuring the fate of cells whose genetic material has been altered and which, inactivated by mutations, can leave the cell proliferation pathway open. Other genes, such as RB1, typically have anti-tumor action by arresting the cell in G-phases. However, by acquiring mutations and losing the function of the encoded protein, they leave the way open for tumor proliferation [14]. 

### 2.1. RAS–RAF–MEK–ERK Signaling Pathway

#### 2.1.1. RAS Gene Family

**Figure 3:**
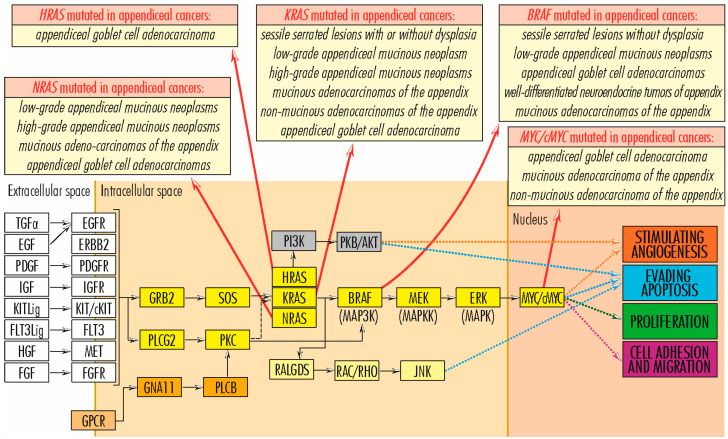
RAS–RAF–MEK–ERK signaling pathway in cancer, pointing the appendiceal cancer types in which members of *RAS* (*KRAS*, *HRAS*, and *NRAS*) and *RAF* (*BRAF*) gene families, and the *MYC/cMYC* gene are mutated (adapted from KEGG Pathways, 2020 [14]).

Of the three members of the *RAS* gene family, in appendiceal cancers, the most common mutations occur in the *KRAS* gene and have an activating effect. The *KRAS* gene (12p12.1) encodes the membrane GTP-ase protein KRAS (189 amino acids; 21,656 Da), which is involved in cell division and proliferation [14]. Inactive in adults, the *KRAS* gene is reactivated by single-amino-acid substitution mutations (missense mutations), and the altered KRAS protein promotes uncontrolled cell proliferation. Regaining *KRAS* gene function due to mutations is common in many tumor types [18], particularly those of the gastrointestinal tract [19,20]. In appendiceal tumors, *KRAS* gene mutations are identified in more than 50% of cases [13], being present in some cases of epithelial tumors: *sessile serrated lesions with* or without dysplasia [15,16,21,22,23], *low-grade appendiceal mucinous neoplasm* (LAMN) [7,8,9,24,25,26,27,28,29,30,31,32,33,34], *high-grade appendiceal mucinous neoplasms* (HAMN) [27,29], *mucinous adenocarcinomas of the appendix* [12,35], *non-mucinous adenocarcinomas of the appendix* [35], and *appendiceal goblet cell adenocarcinoma* [36,37], but is completely lacking in neuroendocrine tumors of the appendix (Figure 3). Among the single-amino-acid substitution (missense) mutations of the KRAS protein identified in appendix cancers are Gly12Asp/Val/Ser/Arg/Cys and Gly13Asp/Arg/Cys [28,29]. In appendiceal cancers, mutations of the other two family members, *HRAS* and *NRAS*, occur much less frequently. The *HRAS* gene (11p15.5), which encodes the HRAS protein (189 amino acids; 21,298 Da), with GTP-ase activity, acquires mutations in Costello syndrome and in several tumor types, such as melanomas, follicular thyroid cancers, bladder cancers, oral squamous cell carcinomas [37,38], and sparsely in certain appendiceal cancers: *mucinous adenocarcinomas of the appendix* and appendiceal adenocarcinomas, especially in *appendiceal goblet cell adenocarcinomas* [9]. Mutations in the NRAS gene (1p13.2), which encodes the NRAS protein (189 amino acids; 21,229 Da), are associated with Noonan and autoimmune lymphoproliferative syndromes, rectal somatic and follicular thyroid cancers, and juvenile myelomonocytic leukemia [39]. *NRAS* gene mutations occur in less than 5% of appendiceal cancers, being present in some cases of low- *and* high-grade appendiceal mucinous neoplasms, in which missense mutation C>A is identified in codon 181, with amino acid replacement Gln61Lys [29], in mucinous adenocarcinomas of the appendix [23] and appendiceal goblet cell adenocarcinomas [26,37]. Raghav and colleagues performed a retrospective review of 607 patients with appendiceal adenocarcinomas. A total of 149 patients underwent molecular testing for activating mutations in *KRAS*, protein expression of c-KIT or COX-2, or microsatellite instability (MSI) status by immunohistochemistry. COX-2 expression and *KRAS* mutations were seen in 61% of patients. High MSI was seen in 6% of patients. COX-2 expression and the presence of *KRAS* mutation did not impact overall survival. Notably, *KRAS* mutations were seen more commonly in well or moderately differentiated tumors than poorly differentiated histology ones. Additionally, clinical–pathologic variables such as age, histologic grade, stage, signet ring cells, and completeness of cytoreduction score are significant prognostic factors for appendiceal adenocarcinomas [40]. Liu et al. conducted a clinical study including 535 patients diagnosticated with colorectal cancer to compare the expression of immune-related genes and the abundance of tumor-infiltrating immune cells in the tumor microenvironment between *KRAS*-mutant and *KRAS* wild-type patients. They observed that NF-κB and T-cell receptor signaling pathways were significantly inhibited in *KRAS*-mutant CRC patients. They concluded that *KRAS* mutation in CRC was associated with suppressed immune pathways and immune infiltration [41]. Matas et al. investigated 24 patients without colorectal adenocarcinoma (CRC) undergoing colonoscopic screening or surveillance and 23 patients with a newly diagnosed primary invasive colorectal adenocarcinoma undergoing surgical resection. They revealed that the normal colon of patients with and without CRC carry mutations in common colorectal cancer genes, but these mutations are more abundant in patients with cancer. Oncogenic *KRAS* mutations were observed in the normal colon of about one-third of patients with CRC but in none of the patients without CRC. Most mutations in the normal colon were different from the driver mutations in tumors, suggesting that independent clones with pathogenic *KRAS* mutations are a common event in the colon of individuals who develop CRC [42].

#### 2.1.2. RAF Gene Family

The *RAF* (rapidly accelerated fibrosarcoma) gene family comprises three independent mammalian genes, Raf-1/c-Raf, B-Raf, and A-Raf, which use MEK1/2 kinases as substrates [43]. In intrauterine life, RAF family members are very active, supporting the growth and development of the embryo and fetus. However, as intense growth processes slow down in extrauterine life, their activity is significantly reduced, activated only by mutations, as in various tumorigenic processes. Of the *RAF* gene family, in appendiceal cancers, the most mutated gene is the *BRAF* (rapidly accelerated fibrosarcoma B) gene (7q34). It encodes the serine/threonine kinase BRAF (766 amino acids; 84,437 Da) and frequently undergoes the missense mutation Val600Glu, which contributes to its activation and being associated with cardiofaciocutaneous, Noonan, and Costello syndromes, as well as with several cancers, including non-Hodgkin’s lymphoma, colorectal cancer, thyroid carcinoma, non-small-cell lung carcinoma, hairy cell leukemia, and adenocarcinoma of the lung [44]. In tumors of the appendix, the most common *BRAF* gene mutation is V600E and it is found in less than 5% of cases of sessile serrated lesions without dysplasia [22], low-grade appendiceal mucinous neoplasm*s* [23], appendiceal goblet cell adenocarcinomas [37], well-differentiated neuroendocrine tumors of appendix [37], and mucinous adenocarcinomas of the appendix. In the X chromosome exists a pseudogene of *BRAF*. Ueda et al. conducted a prospective observational study including 215 patients with right-side colon cancer. *BRAF*V600E mutations of cancer tissue and plasma were detected using droplet digital PCR. They identified *BRAF*V600E mutations in right-side colon cancer at high frequency [45]. Asako et al. performed a retrospective cohort study of the medical records and frozen tissue samples of 268 consecutive patients with stage I-III CRC. This study aimed to investigate the relationship of RAS mutations on an exon basis with clinicopathological features and prognosis in CRC. The RAS mutation rate was significantly associated with age and histology. Patients with KRAS exon 2 mutations exhibited shorter recurrence-free survival than those with KRAS wild-type, KRAS exon 3 mutations, KRAS exon 4 mutations, and NRAS mutation [46].

#### 2.1.3. EGFR/ERBB Gene Family

The *EGFR/ERBB* (*Receptor tyrosine-protein kinase erbB*) gene family comprises four members (*EGFR/ERBB1*—*Receptor tyrosine-protein kinase erbB-1*, *HER2/ERBB2*—*Receptor tyrosine-protein kinase erbB-2*, *HER3/ERBB3*—*Receptor tyrosine-protein kinase erbB-3* and *HER4/ERBB4*—*Receptor tyrosine-protein kinase erbB-4*), with tyrosine kinase function and structurally related to epidermal growth factor receptor (EGFR). Anchored in the plasma membrane, EGFR/ERBB family members transmit signals via the RAS–RAF–MEK–ERK, PI3K–PKB/AKT, JAK–STAT, and PLC signaling pathways, sustaining cell proliferation, angiogenesis, and evading apoptosis [14]. Mutated or overexpressed proteins are associated with several types of malignancies of the lung, breast, stomach, head, neck, colorectal cancer, pancreatic carcinomas, glioblastoma, and some appendiceal cancers, with the latter being associated with mutations in *EGFR/ERBB1* (7p11.2), *HER2/ERBB2* (17q12), and *HER4/ERBB4* (2q34) genes, unlike other family members. The EGFR/ERBB1 protein (1210 amino acids; 134,277 Da) is a component of the cytokine storm associated with severe acute respiratory syndrome coronavirus-2 (SARS-CoV-2) [47]. The HER2/ERBB2 protein (1255 amino acids; 137,910 Da) has a not-yet-known ligand, but forms a heterodimer with the kinase-deficient protein HER3/ERBB3, which constitutes the most potent signaling complex in the EGFR/ERBB family [48,49], while the HER4/ERBB4 protein (1308 amino acids; 146,808 Da) causes the formation of kinase-active hetero-oligomers [50,51]. In appendiceal cancers, mutations in the *EGFR/ERBB1* gene are found in *well-differentiated neuroendocrine tumors of the appendix* [37]; mutations in *HER2/ERBB2* gene are associated with the adenocarcinomas of the *appendiceal goblet cells*, *well-differentiated neuroendocrine tumors of the appendix*, and *mucinous adenocarcinomas of the appendix*; and those in the *HER4/ERBB4* gene in *well-differentiated neuroendocrine tumors of the appendix* [37].

#### 2.1.4. MET Gene

**Figure 4:**
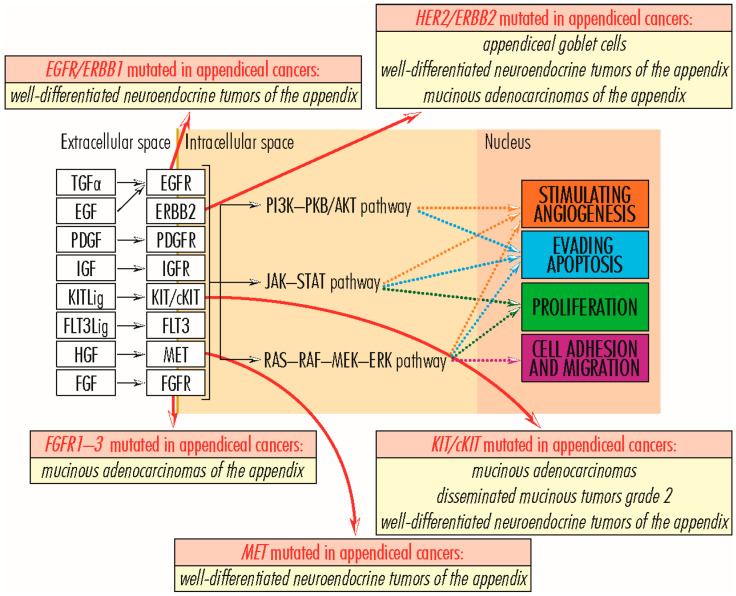
Receptors of PI3K–PKB/AKT, JAK–STAT, and RAS–RAF–MEK–ERK signaling pathways in cancer, pointing out the appendiceal cancer types in which the most important members are mutated (adapted from KEGG Pathways, 2020 [14]).

*MET* gene—*MNNG HOS transforming gene* (7q31.2) encodes the MET protein (1390 amino acids; 155,541 Da), a class IV receptor tyrosine kinase family member, expressed on the surface of epithelial cells. Through interaction with hepatocyte growth factor (HGF), the MET protein dimerizes, becoming active and supporting cell proliferation, cell motility, and invasiveness via the RAS–RAF–MEK–ERK signaling pathway; angiogenesis and invasion via the JAK–STAT signaling pathway; apoptosis evasion and cell survival via the PI3K–PKB/AKT signaling pathway; and embryogenesis. During the embryo–fetal period, the HGF–MET pair is involved in trophoblast formation, liver formation, myoblast migration, kidney development, nerve wiring, small airways segmentation, melanocyte migration, differentiation of erythroid progenitors, and mammary gland formation; after birth, it participates in tissue repair and wound healing, as well as a protective agent against pulmonary fibrosis, cirrhosis, myocardial ischemia, and acute kidney injury. Overexpression of the two proteins is common in solid tumors; MET mutations are associated with papillary renal cell carcinoma, hepatocellular carcinoma, and several types of tumors formed in the head and neck region [14,52,53]. In *low-grade appendiceal mucinous neoplasms*, mutations in the *MET* gene are insignificant and occur rarely [26,30], acquiring greater importance and occurring more frequently in *well-differentiated neuroendocrine tumors of the appendix* [37] (Figure 4). Li et al. conducted a study aiming to assess the clinical utility of TP53 mutation and MET amplification in ctDNA as biomarkers for monitoring advanced gastric cancer disease progression. They performed mutation detection on circulating-tumor DNA in 23 patients with advanced gastric cancer and identified the top 20 mutant genes. The five most frequently mutated genes were *TP53* (55%), *EGFR* (20%), *ERBB2* (20%), *MET* (15%), and *APC* (10%). *TP53* was the most common mutated gene (55%), and *MET* had a higher frequency of mutations (15%). They concluded that *TP53* mutation and *MET* amplification in circulating-tumor DNA could predict the disease progression of advanced gastric cancer patients [54].

#### 2.1.5. FGFR Gene Family

The *FGFR* gene family consists of four transmembrane tyrosine kinase receptors: *FGFR1*—*fibroblast growth factor receptor 1* (8p11.23), which encodes FGFR1 protein (822 amino acids; 91,868 Da); *FGFR2*—fibroblast growth factor receptor 2 (10q26.13), which encodes FGFR2 protein (821 amino acids; 92,025 Da); *FGFR3*—*fibroblast growth factor receptor 3* (4p16.3), which encodes FGFR3 protein (806 amino acids; 87,710 Da); and *FGFR4—fibroblast growth factor receptor 4* (5q35.2), which encodes FGFR3 protein (802 amino acids; 87,954 Da), with highly conserved structure between members during evolution, but with differences in ligand affinity and tissue distribution. FGFRs have 22 ligands; activate the RAS–RAF–MEK–ERK, JAK–STAT, PI3K–PKB/AKT, and PLC signaling pathways; and are involved in cell proliferation and survival by evading apoptosis and in stimulating angiogenesis [14,55]. Genomic alterations of *FGFR*s (single-nucleotide variants, gene fusions, and copy number amplifications) are identified, on average, in 5–10% of cancers, with higher frequencies of 10–30% in urothelial cancer and intrahepatic cholangiocarcinoma. Single-nucleotide variations occur less frequently in the *FGFR1* gene than other family members and have missense and activating effects when they occur in the kinase domains (Met546Lys and Lys656Glu) or unknown when they affect the Ser125Leu locus. In the *FGFR2* gene, single-nucleotide variants express mutations with an activating effect more frequently in the transmembrane (Tyr375Cys, Cys382Tyr/Arg) and extracellular domains (Ser252Trp, Trp290Cys, Pro253Arg) and more rarely in the kinase domain (Asn549His/Lys, Lys659Glu). In the *FGRF3* gene, activating mutations are present in 10 to 60% of urothelial cancers and about 5% of cervical cancers, more frequently in low-grade tumors and often affecting the extracellular domain (Arg248Cys and Ser249Cys) and the transmembrane domain (Gly370Cys and Tyr373Cys). In contrast, in the *FGFR4* gene, missense mutations Val550Glu and Asn535Lys occur in approximately 7–8% of rhabdomyosarcoma cases, causing autophosphorylation and constitutive activation of the kinase domain, and Tyr367Cys in the human breast cancer cell line, MDA-MB453, in which constitutive activation of the kinase domain causes receptor activation in a ligand-independent manner. Gene fusions of *FGFR* family members (*FGFR1*–*3*) with various other partner genes are identified in several cancers, including breast, carcinomas, and adenocarcinomas. Most copy number amplification events occur in the FGFR1 gene [49], being associated with several cancers, particularly breast cancers, to which they indicate poor prognosis and recurrence, and *FGFR4* genes [56], but they are also present in other family members, with those of *FGFR2* gene being found in gastric and breast tumors [57,58]. In appendiceal cancers, mutations in FGFR1–3 genes are present in mucinous adenocarcinomas of the appendix [59]. Jogo et al. examined whether ctDNA helps detect *FGFR2* amplification and co-occurring resistance mechanisms in advanced gastric cancer in a nationwide ctDNA screening study. In addition, they examined patients with *FGFR2*-amplified advanced gastric cancer identified by ctDNA sequencing who received *FGFR* inhibitors. *FGFR2* amplification was more frequently detected by ctDNA sequencing in 28 (7.7%) of 365 patients with advanced gastric cancer than by tissue analysis alone (2.6–4.4%). *FGFR2* amplification profiling of paired tissue and plasma revealed that *FGFR2* amplification was detectable only by ctDNA sequencing in 6 of 44 patients, which was associated with a worse prognosis. The researchers observed that ctDNA sequencing identifies *FGFR2* amplification missed by tissue testing in patients with advanced gastric cancer [60].

#### 2.1.6. KIT/cKIT Gene

*KIT/c-KIT* gene—V-Kit Hardy-Zuckerman 4 Feline Sarcoma Viral Oncogene-Like Protein (4q12) encodes KIT/cKIT protein (976 amino acids; 109,865 Da), with function as a membrane tyrosine kinase receptor and involved in the transduction of the activating signal for cell proliferation, via the RAS–RAF–MEK–ERK signaling pathway, for cell survival by evading apoptosis, via the PI3K–PKB/AKT and PLC signaling pathways, and for angiogenesis, via the JAK–STAT and RAS–RAF–MEK–ERK signaling pathways [14]. *KIT/c-KIT* gene activation is oncogenic and associated with several tumor types, such as melanoma, lung cancer, and gastrointestinal stromal cancers [37]. In cancers of the appendix, *KIT/c-KIT* gene activating mutations occur rarely and are identified in a small number of cases of mucinous adenocarcinomas [12] and in disseminated mucinous tumors grade 2, whereas in well-differentiated neuroendocrine tumors of the appendix, mutations in the *KIT/c-KIT* gene appear to have a higher frequency [37].

#### 2.1.7. MYC/c-MYC Gene

The *MYC/c-MYC* gene (8q24.21) encodes the MYC/c-MYC protein (439 amino acids; 48,804 Da), with essential functions in cell growth and cell metabolism. Through the RAS–RAF–MEK–ERK signaling pathway, the MYC/c-MYC protein is involved in cell proliferation and angiogenesis; through the WNT, ESR/JUP–FOS/JUN/SP1/NCOA/ESR, and MYC/c-MYC–CCND–RB1–E2F signaling pathways, it is involved in cell proliferation; and through MYC/c-MYC–E2F signaling pathway, it plays a role in blocking cell differentiation [14,61], which are very important in the progression of tumor processes. On the other hand, the MYC/c-MYC protein appears to induce apoptosis, which counteracts its pro-tumor effects. In non-transformed cells, *MYC/c-MYC* gene expression is regulated by developmental factors or mitogenic signals and the short lifespan of mRNA and MYC/c-MYC protein. However, in several cancers, *MYC/c-MYC* genes and those downstream of signaling pathways that stimulate cell proliferation almost always become overexpressed through mutations in the *MYC/c-MYC* gene or, more commonly, by induction of its expression due to activation of an upstream gene (e.g., *ERK*), or by its life extension due to mutations, such as that in the Thr58 phosphorylation site, which reduce the efficiency of its phosphorylation-dependent ubiquitination. This process is commonly seen in human lymphomas. Mutations in the *MYC/c-MYC* gene, which presumably have a stimulatory effect on its function, are also reported in less than 10% of cases of appendiceal goblet cell adenocarcinoma *and* mucinous/non-mucinous adenocarcinoma of the appendix [62,63], being a secondary mutation in appendiceal cancers. Lin et al. performed a retrospective analysis including 494 patients with breast cancer. Genomic alterations were determined using next-generation sequencing. Survival analysis was applied to assess the effects of genetic alterations on relapse-free survival. Additionally, they used logistic regression to identify the factors associated with pathological complete response after neoadjuvant chemotherapy. Patients with *TP53/MYC* co-alteration exhibited higher grade and stage, more positive HER2 status, and higher Ki67 levels but fewer luminal A subtypes. They also had more mutations in genes involved in ERBB and TGF-β signaling pathways, as well as exclusive FANCG/CDKN2B/QKI copy number amplifications and SUFU/HIST3H3/ERCC4/JUN/BCR mutations. Concurrent *TP5*3 and *MYC* alterations independently increased relapse hazards, conferring unfavorable prognoses [64].

#### 2.1.8. PLCG2 Gene

*PLCG2* gene (16q24.1) encodes PLCγ2–*Phospholipase C Gamma 2 protein* (1265 amino acids; 147,870 Da), a transmembrane signaling enzyme, which, in the presence of calcium ions, catalyzes the transition of 1-phosphatidyl-1D-myo-inositol 4, 5-bisphosphate to 1D-myo-inositol 1, 4, 5-trisphosphate (IP3) and diacylglycerol (DAG), which function as secondary messengers for growth factors (TGFα/EGF to EGFR/ERBB1 and PDGF to PDGFR) and transmembrane immune receptors for various cellular functions, including proliferation and angiogenesis; through the RAS/RAF–MEK–ERK signaling pathway, proliferation and evading apoptosis; and through RAS–PI3K pathway, endocytosis and calcium efflux [14]. *PLCG2* gene dysfunctions are associated with several pathologies, including neurodegenerative diseases, immune disorders (familial cold autoinflammatory syndrome three and antibody deficiencies), and cancer [65,66]. Peng and colleagues investigated a 7-year-old patient with APLAID, a rare primary immunodeficiency caused by gain-of-function mutations in the *PLCG2* gene. The patient carried a novel de novo missense mutation c.2534T>C in exon 24 of the *PLCG2* gene that causes a leucine-to-serine amino acid substitution (p.Leu845Ser). Bioinformatics analysis revealed that this mutation harmed the structure of the PLCγ2 protein, which is highly conserved in many other species. Also, immunophenotyping by flow cytometry revealed that in addition to the typical decrease in circulating memory B cells, the levels of myeloid dendritic cells (mDCs) in the children’s peripheral blood were significantly lower, as were the CD4+ effector T cells induced by their activation [67]. Thus, in solid colon tumors and soft tissue sarcoma, *PLCG2* gene expression positively correlates with immune cell infiltration into the tumor microenvironment and favorable prognosis [68], while *PLCG2* gene mutations produce resistance of some tumors to chemotherapy. In appendiceal tumors, a small number of cases carry mutations in the *PLCG2* gene with no reference to their effect [37]. 

### 2.2. TP53 Signaling Pathway

**Figure 5:**
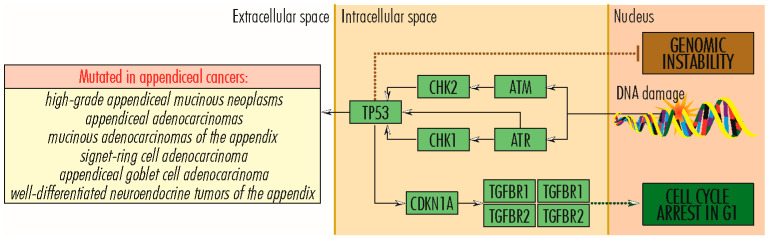
TP53 signaling pathway in cancer, pointing out the appendiceal cancer types in which *TP53* gene is mutated (adapted from KEGG Pathways, 2020 [14]).

The *TP53* gene (17p13.1) encodes P53–*protein 53* or TP53–*tumor protein 53* (393 amino acids; 43,653 Da), which, because of the essential functions it performs in the proper functioning of cells, is nicknamed *the guardian of the genome*. Protein 53 occupies a central place in the TP53 signaling pathway (Figure 5). It plays a vital role in protecting DNA integrity and regulating DNA repair in cell division, differentiation, and senescence; regulating metabolism; and suppressing tumor development. Protein 53 is a transcription factor located in the nucleus of all cells in the body, attaches directly to genetic material, is activated by cellular stress signals (oncogene activation, DNA damage, DNA replication stress), controls stress-specific gene transcription, and is involved in determining cell fate [69,70] and in signaling pathways in cancer, in which the relationship with HIF1A is essential. *HIF1A* gene expression is induced by reduced intracellular oxygen pressure, and the HIF1A protein causes *TP53* activation, which plays a role in stopping the tumor process. TP53 is degraded in proteasomes by a mechanism ubiquitin-dependent/independent mediated by MDM2–*Human Homolog of Mouse Double Minute 2*. Mutations in the *TP53* gene, which lead to abnormal protein synthesis, are found in various cancers, including Li Fraumeni syndrome, which predisposes to tumors occurring at a young age [70]. In appendiceal tumors, *TP53* mutations rarely occur in *low-grade appendiceal mucinous neoplasms* [23], in which they are a marker of progression to high-grade tumors, and *high-grade appendiceal mucinous neoplasms* [23,33], and with higher frequency in *appendiceal adenocarcinomas*, *mucinous adenocarcinomas of the appendix* [16,23,33], *signet ring cell adenocarcinoma* [33], and *appendiceal goblet cell adenocarcinoma* [37], whose aggressive properties are due, to a reasonable extent, to *TP53* inactivation by mutations [33], as well as in *well-differentiated neuroendocrine tumors of the appendix* [33]. Oka and colleagues investigated a case of early appendiceal adenocarcinoma in a female who presented at the hospital because of an enlarged appendix noted by contrast-enhanced CT performed for hematuria. They observed that the atypical epithelium in a small area at the tip was particularly strong in nuclear atypia and showed a strong positive diffusely in p53, which was an image of well-differentiated tubular adenocarcinoma [71]. Yanai et al. investigated 51 appendiceal mucinous tumors using a next-generation sequencing (NGS) cancer hotspot panel. They detected *p53* mutation in 31 cases, mainly in low-grade mucinous appendiceal neoplasms [72]. Yan and colleagues determined the p53 levels in pseudomyxoma peritonei of appendiceal origin and correlated the levels with clinicopathological characteristics and overall survival in 141 patients. They concluded that the analysis of aberrant *p53* might provide the basis for evaluating the biological behavior of pseudomyxoma peritonei and predicting clinical outcomes [73].

### 2.3. PI3K–PKB/AKT Signaling Pathway

#### 2.3.1. *AKT1* Gene

The *AKT1* gene (14q32.33) encodes the AKT1–*AKT Serine/Threonine Kinase 1 protein* (480 amino acids; 55,686 Da), one of the core pillars of the PI3K–PKB/AKT signaling pathway and its most active member. Unlike other signaling pathways, PI3K–PKB/AKT is often activated by gene mutations encoding its proteins, triggering cascade signaling for cell proliferation and survival by preventing apoptosis and angiogenesis. The AKT1 protein receives signals from the PI3K, which in turn transmits signals from numerous proteins, including RAS, which links to the RAS/RAF–MEK–ERK signaling pathway, and further transduces them to numerous targets [14,74,75]. *AKT1* gene mutations occur with some frequency in cancer, particularly the one in the pleckstrin homology domain, Glu17Lys, which enhances its binding to the PI3K ligand and relocalization to the plasma membrane, intensifying cell migration and resistance to chemotherapeutic treatment in breast cancers, as well as selective destruction of chemotherapy-resistant tumor cells and those with low levels of *AKT* gene expression [75,76,77]. Activating mutations of the *AKT1* gene (Glu17Lys and Glu49Lys) are associated in a small number of cases with breast cancers, head and neck squamous cell carcinomas, endometrial cancers, non-small-cell lung cancers, renal cancers, and appendix cancers, the latter of which are occasionally reported *in* low-grade appendiceal mucinous neoplasms [76].

#### 2.3.2. ITGA11 Gene

The *ITGA11* gene (15q23) encodes the ITGA11–*Integrin Subunit Alpha 11 protein* (1188 amino acids; 133,470 Da) forms a heterodimer with the beta subunit, binding collagen, and is involved in muscle cells attaching to the extracellular matrix, as well as transducing signals for cell proliferation, evasion of apoptosis, and enhancement of angiogenesis, mainly through the PI3K–PKB/AKT signaling pathway [14]. Also, overexpression of the ITGA11 gene in fibroblasts associated with breast cancer and non-small-cell lung cancers indicates high grades and poor prognosis [78,79,80]. On the other hand, mutations in the ITGA11 gene have been reported only occasionally *in* appendiceal mucinous neoplasms, with no correlation with the degree of differentiation or tumor progression [7].

#### 2.3.3. PIK3C2B and PIK3CA Genes

**Figure 6:**
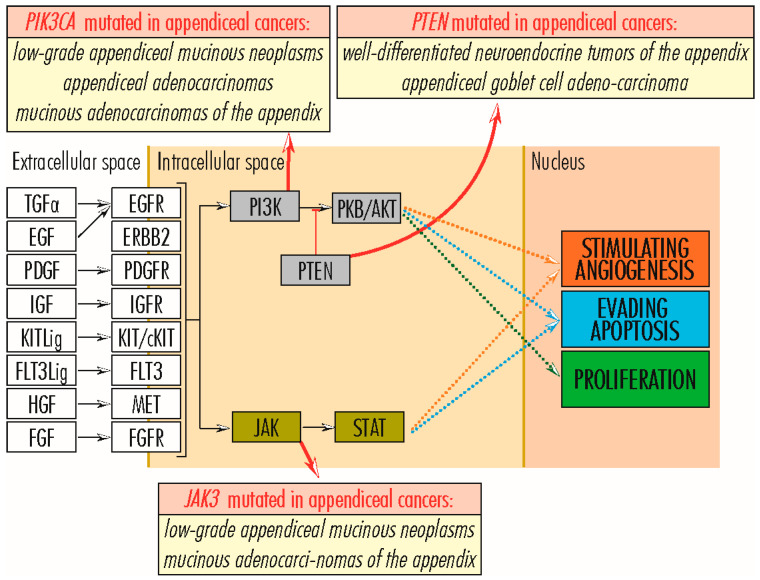
PI3K–PKB/AKT and JAK–STAT signaling pathways in cancer, pointing out the appendiceal cancer types in which *PIK3CA*, *PTEN*, and *JAK3* genes are mutated (adapted from KEGG Pathways, 2020 [14]).

*PIK3C2B* (1q32.1) and *PIK3CA* (3q26.32) genes encode two phosphoinositide 3-kinases (PI3Ks), PIK3C2B–*Phosphatidylinositol-4-Phosphate 3-Kinase Catalytic Subunit Type 2 Beta* (1634 amino acids; 184,768 Da) and PIK3CA–*Phosphatidylinositol-4, 5-Bisphosphate 3-Kinase Catalytic Subunit Alpha* (1068 amino acids; 124,284 Da), with catalytic activity and phosphorylating inositol lipids. They are important nodes in the PI3K–PKB/AKT signaling pathway, integrating biological signals from a wide variety of sources, including the entire range of membrane receptors of the RAS/RAF–MEK–ERK signaling pathway, directly or via the RAS protein, or FAK/PTK2–*protein tyrosine kinase 2*, a ligand of membrane integrins, and further transducing them to the PKB/AKT protein, to stimulate cell proliferation and angiogenesis, and being involved in cell survival by evading apoptosis [14,81]. Mutations in the *PIK3C2B* gene, including Arg564Cys, occur in a wide variety of cancers [82], such as endometrial, breast, ovarian, colorectal, bladder, lung, cervical, head and neck, prostate, esophageal, liver, and renal cancers [83]; in a case of *low-grade appendiceal mucinous neoplasms;* and some cases of glioblastoma, melanoma, sarcoma, and megalencephaly. In leukemias, increased expression of the PIK3C2B gene can lead to resistance to chemotherapeutic agents [84]. On the other hand, activating mutations of *the PIK3CA* gene shift tumor behavior, enhancing cell proliferation and migration and tumor invasiveness and metastasis [84], being reported in breast cancers [83], colorectal cancers, head and neck squamous cell carcinoma, and appendiceal cancer, including low-grade appendiceal mucinous neoplasms [12,30], appendiceal adenocarcinomas [85], and mucinous adenocarcinomas of the appendix [33]. Activating mutations of *PIK3CA* are correlated with poor prognosis, even in patients whose tumor is wholly excised [86]. Also, in a wide variety of neoplasms, *PIK3C* genes appear amplified, whereas in prostate cancer, the *PIK3CA* gene is overexpressed [85,87] (Figure 6).

#### 2.3.4. PTEN Gene

*PTEN* gene (10q23.31) encodes PTEN–*phosphatase* and tensin homolog protein (403 amino acids; 47,166 Da), with antitumor function by preferentially dephosphorylating 3-position on inositol head groups and reversing the reaction catalyzed by PI3Ks, inhibiting cell proliferation and migration and tumor invasiveness and metastasis, by inactivating the transmission of biological signals through the PI3K–PKB/AKT pathway [14,86,88]. Mutations and inactivation of the *PTEN* gene frequently occur in numerous tumors, including *well-differentiated neuroendocrine tumors of the appendix* [37] and *appendiceal goblet cell adenocarcinoma*, sometimes in association with activating mutations in the *PIK3CA* gene [86].

### 2.4. AKT-mTOR Signaling Pathway

The *CDKN1B* (12p13.1) and *CDKN2A* (9p21.3) genes encode the CDKN1B–*Cyclin-Dependent Kinase Inhibitor 1B* (198 amino acids; 22,073 Da) and CDKN2A–*Cyclin-Dependent Kinase Inhibitor 2A* (132 amino acids; 13,903 Da) proteins. CDKN1B protein inhibits the activity of cyclin CDK2 bound to cyclin A or E; inhibits or activates cyclin D–CDK4 complexes concerning microenvironment variables; contributes to the realization, stabilization, modulation, and activation of the CCND1–CDK4 complex; and inhibits cell proliferation, by blocking the cell cycle at G1 stage. Mutations in the *CDKN1B* gene occur only in some types of cancers, including breast, prostate, and small intestine neuroendocrine tumors [14,89,90] and *appendiceal goblet cell adenocarcinoma*. On the other hand, the *CDKN2A* gene encodes two transcripts, p16INK4A and p14ARF, with differences in the first exons, inhibiting CDK4 activity, being involved in stopping the degradation of TP53 protein by the E3 ubiquitin-protein ligase MDM2, to which it binds and sequesters. Blocking the cell cycle at the G1 stage, the CDKN2A protein inhibits cell proliferation [14,91,92]. In many types of cancers, such as adenocarcinomas of the uterus, bladder, stomach, colorectal, cervical, brain and papillary thyroid cancers, head and neck squamous cell carcinoma, malignant peripheral nerve sheath tumor, neurilemmoma, pituitary macroadenoma, multiple myeloma, neuroblastoma, osteochondroma, low-grade neuroepithelial tumor, non-Hodgkin’s lymphoma, glioblastoma multiforme, astrocytoma, meningioma [91], *low-grade appendiceal mucinous neoplasms*, *appendiceal goblet cell adenocarcinoma*, and *well-differentiated neuroendocrine tumors of the appendix* [37], a wide variety of inactivating mutations are reported. 

### 2.5. RB1 Signaling Pathway

The *RB1* gene (13q14.2) encodes the RB1–*Retinoblastoma-Associated Protein* or *RB Transcriptional Corepressor 1 protein* (928 amino acids; 106,159 Da), which, by methylating histones and stabilizing heterochromatin, maintains the overall chromatin structure and negatively regulates the cell cycle, preventing cell proliferation. In the dephosphorylated or hypo-phosphorylated state, RB1 protein interacts with members of the E2F–*Retinoblastoma-Associated Protein 1* family and blocks transcription of many of E2F-responsive genes required for progression through S-phase, mitosis, and cytokinesis, thus preventing G1/S transition of the cell cycle. Phosphorylation mediated by cyclin-dependent kinases CDK4/6 and dissociation of the RB1–E2F heterodimer by their and cyclins activity leads to inactivation of RB1 and expression of downstream genes that determine cell progression through the S phase. Through phosphorylation and CDK3/cyclin C-induced activation, RB1 initiates the G0-G1 transition. The degradation of RB1 in proteasomes in a ubiquitin-dependent and -independent manner is promoted by MDM2–*Human Homolog of Mouse Double Minute 2*. The loss of *RB1* gene function through mutation or other mechanisms leads to retinoblastoma, rare cancer that is the primary type of ocular neoplasia in children and is also found in small cell cancer of the lung, endometrial cancer, bladder cancer, and osteogenic sarcoma [14,93,94,95,96]. In appendiceal cancers, *RB1* gene mutations are reported in some isolated cases of appendiceal cancers without further specification, *low-grade appendiceal mucinous neoplasms* [15,30], *mucinous adenocarcinomas of the appendix*, and *appendiceal goblet cell adenocarcinoma*, without being defined for them.

### 2.6. JAK3 Signaling Pathway

The *JAK3* gene (19p13.11) encodes the JAK3–*Janus Kinase 3 protein* (1124 amino acids; 125,099 Da), a critical JAK–STAT signaling pathway member. The JAK3 protein collects signals from many cytokine receptors, including activating RAS/RAF–MEK–ERK signaling pathway receptors. It transmits them to VEGFs (*Vascular Endothelial Growth Factors*) via STAT (*Signal Transducer and Activator of Transcription*) proteins to amplify angiogenic processes [14]. The *JAK3* gene is predominantly expressed in immune system cells. Its underexpression is associated with autosomal SCID (*Severe Combined Immunodeficiency Disease*) [97] and oncogenic activation with natural killer/T-cell lymphoma, a rare and aggressive non-Hodgkin’s lymphoma [98], and in malignant T cells in cutaneous T cell lymphoma [99]. In appendiceal cancers, *JAK3* gene mutations occur occasionally and are reported in *low-grade appendiceal mucinous neoplasms* [15,30] and *mucinous adenocarcinomas of the appendix*.

### 2.7. Wnt/β-Catenin Signaling Pathway

#### 2.7.1. APC Gene

The *APC* gene (5q22.2) encodes the APC–*Adenomatosis Polyposis Coli Tumor Suppressor protein* (2843 amino acids; 311,646 Da), with antagonistic regulatory function of the WNT signaling pathway [14,100]. The WNT signaling pathway is actively involved in tissue morphogenesis and repair and is physiologically functional in the embryo–fetal stage. In the adult, it is blocked through β-catenin inactivation due to the formation of a complex between GSK3–*Glycogen Synthase Kinase-3*, APC–*Adenomatosis Polyposis Coli*, AXIN–*Axis Inhibitor*, and CKIa–*Casein Kinase Ia* [17], with mutations that inactivate genes encoding proteins of the complex being likely to inhibit its formation and allow the canonical (β-catenin-dependent) WNT signaling pathway to function. In blocking the WNT signaling pathway, APC protein plays a vital role in inhibiting cell proliferation and survival (by evading apoptosis); tumor processes stimulated by it; and cell migration, adhesion, differentiation, and chromosome segregation [82]. Mutations in the *APC* gene inhibit its function and are generally associated with cancers of the digestive tract, such as stomach, colon/colorectal [101,102], and some appendiceal cancers, such as low-grade appendiceal mucinous neoplasms, appendiceal adenocarcinomas [16,33,85], mucinous adenocarcinomas of the appendix, and appendiceal goblet cell adenocarcinoma [30].

#### 2.7.2. AXIN1 Gene

The *AXIN1* gene (16p13.3) encodes the AXIN1–*Axis Inhibitor 1 or Axis Inhibition Protein 1 protein* (862 amino acids; 95,635 Da), which, together with *APC*, *GSK3*, and *CKIa*, enters the structure of the complex to inactivate β-catenin and thereby block the WNT signaling pathway, inhibiting cell proliferation and survival, by evading apoptosis [14,103]. Inactivating mutations in the *AXIN1* gene are reported in hepatocellular carcinoma, hepatoblastomas, ovarian endometrioid adenocarcinomas, medulloblastomas [103], prostate cancers [104], and colorectal cancers [105]. In appendiceal cancers, the missense mutation (Arg484Cys) is reported in a case of low-grade appendiceal mucinous neoplasm with no known significance.

#### 2.7.3. TCF7L2 Gene

The *TCF7L2* gene (10q25.2-q25.3) encodes the transcription factor TCF7L2–*Transcription Factor 7-Like 2 (T-Cell Specific, HMG-Box)* (619 amino acids; 67,919 Da) in intestinal cells. It comprises a high-mobility group (HMG). It plays an essential role in transmitting mitogenic signals required for cell proliferation and those required for cell survival by inhibiting apoptosis. TCF7L2 is an essential node in the WNT signaling pathway, receiving signals from β-catenin and positively regulating the *MYC/c-MYC* proto-oncogene and the BIRC5–*Baculoviral IAP Repeat Containing five proteins*. TCF7L2 protein also blocks the cell cycle inhibitors CDKN2C/CDKN2D. On the other hand, TCF7L2 manifests an anti-tumor function by suppressing cell motility and invasiveness and by directly suppressing the activity of the pro-metastatic RUNX2–*Runt-related transcription factor 2*, its loss of function stimulating tumor malignancy [14,106,107]. TCF7L2 protein is also involved in blood glucose homeostasis, with some defects in it associated with an increased risk of developing type 2 diabetes or nonspecific syndromic intellectual disability [106]. Polymorphisms of this gene are reported in colorectal cancer [107,108] and a small fraction of appendiceal cancers without specifying their type.

## 3. Mutations in Genes Whose Proteins Are Involved in Cell Survival and Tumor Invasiveness/Metastasis

The survival of transformed cells, through insensitivity to apoptotic signals and escape from programmed cell death, is essential for tumor progression and is ensured through several signaling pathways, including RAS–RALGDS–RHO–JNK, in which *RHO* family genes and PI3K–PKB/AKT play an important role. On the other hand, *RHO* family members are involved in tumor invasiveness and metastasis in angiotensinogen-initiated signaling pathways [14].

### RHOA Gene

The *RHOA* gene (3p21.31) encodes the RHOA–*Ras Homolog Gene Family protein, Member A* (193 amino acids; 21,768 Da), a member of the RHO family of small GTPases that function as molecular switches in various signaling pathways, including MEMO1–RHOA–DIAPH1, WNT11, and RAS–RAF–MEK–ERK. RHOA protein stimulates actin filament reorganization in the cytoskeleton and regulates cell adhesion and motility. Via the JNK protein, it promotes cell survival by evading apoptosis, and via ROCK–*Rho protein associated coiled-coil containing protein kinases*, by evading tumor invasiveness and metastasis [109]. Overexpression and mutations of this gene are associated with tumor cell proliferation and metastasis in germinal center-derived B cell lymphomas, angioimmunoblastic T cell lymphoma, and gastric adenocarcinomas [110]; invasiveness and poor prognosis in colorectal cancers [111]; and reduced *RHOA* gene expression, in parallel with amplified signaling by the cytokines CCR5 and CXCR4, is associated with breast cancer metastasis [112]. It is unclear whether the RHOA protein functions as a promoter or suppressor of tumorigenesis. In appendiceal cancers, two possible pathogenic mutations (Tyr42Cys and Asp49His) are identified in a few cases of *appendiceal goblet cell adenocarcinoma*.

## 4. Mutations in Genes Regulating the Angiogenesis

Angiogenesis is the main mechanism by which tumors larger than 2–4 mm in diameter are supplied with oxygen and nutrients. The main signaling pathways by which new blood vessel formation is stimulated from pre-existing ones are GNA11–PLCβ–RAS–RAF–MEK–ERK, BCR/ABL/JAK–STAT–VEGF, PI3K–PKB/AKT, β-catenin–TCF/LEF, NOTCH, and HIF–VEGF–TGF [14].

### 4.1. GNA11 and GNAS Genes

**Figure 7:**
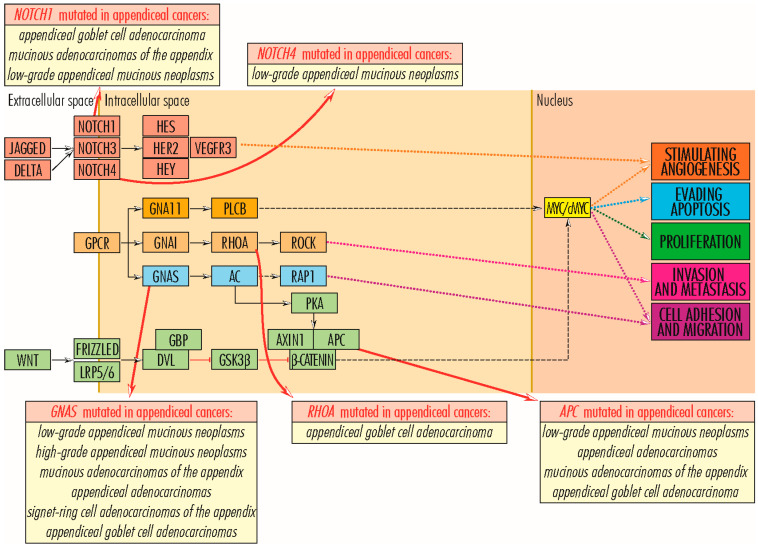
WNT, angiogenesis, and NOTCH signaling pathways in cancer, pointing out the appendiceal cancer types in which *GNAS*, *RHOA*, *NOTCH1*, *NOTCH4*, and *APC* genes are mutated (adapted from KEGG Pathways, 2020 [14]).

The *GNA11* and *GNAS* genes encode *guanine nucleotide-binding proteins* (G proteins), which modulate or transmit many biological signals and consist of three units: alpha, beta, and gamma. The GNA11–*Alpha subunit 11* (359 amino acids; 42,123 Da), encoded by the *GNA11* gene (19p13.3), activates the beta subunit of phospholipase C, further transmitting signals to RAS and RAF proteins in the RAS/RAF–MEK–ERK signaling pathway, whereby it stimulates cell proliferation and survival by evading apoptosis, and angiogenesis via the RAS–RAF–MEK–ERK pathway [14,113]. The 626A>C (Gln209Pro) and 627G>C (Gln209His) mutations are reported in some benign hemangiomas [114] and a case of *mucinous adenocarcinoma of the appendix*. On the other hand, the *GNAS* gene (20q13.32) shows a complex expression pattern with several alternatively spliced variants, one of which forms the ubiquitously expressed GNAS–*G Protein Subunit Alpha S* or *Secretogranin VI* (626 amino acids; 67,948 Da), with an essential function in the action of numerous hormones and other molecules and involved in cAMP activation, which in turn links to several signaling pathways. Thus, the *GNAS* gene product indirectly stimulates, via PKA–*protein kinase A*, the formation of the AXIN1–APC complex in the WNT signaling pathway, with an inhibitory function on β-catenin and cell proliferation and promoting cell death through apoptosis. Also, through RAPGEF2–*Rap guanine nucleotide exchange factor 2*, GNAS links the cAMP pathway to the RAS/RAF–MEK–ERK signaling pathway, promoting cell proliferation and angiogenesis, and through phospholipase C, GNAS participates in the activation of RAP1–*Ras-related protein Rap-1A*, involved in gene activation and cell proliferation, survival, adhesion, migration, and polarity. The *GNAS* gene is biallelically expressed in most tissues and is specific-paternally silenced in a small number of tissues, leading to differential transmission of parental phenotypes in *GNAS* mutations [115,116]. Mutations at this locus are associated with pseudohypoparathyroidism (PHP) type I, progressive osseous heteroplasia, McCune–Albright Syndrome, and breast cancer, with no known role in their progression [116,117]. In appendiceal cancers, *GNAS* mutations are reported with high frequency in *low*- and *high-grade appendiceal mucinous neoplasms* [15,16,30,33], in codons 601 (c.601 C>T, p. Arg201His) and 602 (c.602 G>A, p. Arg201Cys), in which they often occur together with mutations in the *KRAS* gene as early as G1 and G2 [15] stages and are associated with abundant mucin production, without affecting patient survival [27], and in *mucinous adenocarcinomas of the appendix*. With reduced frequency, mutations in the *GNAS* gene also occur in *appendiceal adenocarcinomas*, *signet ring cell adenocarcinomas of the appendix*, and *appendiceal goblet cell adenocarcinomas* (Figure 7).

### 4.2. EP300 and CPB/CREBBP Genes

The *EP300* (22q13.2) and *CBP/CREBBP* (16p13.3) encode two histone acetyltransferase (HAT) paralogs, EP300–*E1A Binding Protein P300* (2414 amino acids; 264,161 Da) and CBP/CREBBP–*Cyclic adenosine monophosphate Response Element Binding Protein* or *CREB-binding protein* (2442 amino acids; 265,351 Da), which act as cofactors in stimulating the transcription of numerous genes, including the *MYB*, *JUN*, *FOS*, *E1A*, and *E6* oncogenes, and the tumor-suppressor proteins TP53, E2F, RB, SMADs, RUNX, and BRCA1 [118]. EP300 and CBP/CREBBP act mainly on histone H3, which acetylates the lysine residue at position 27 (H3Lys27ac), and, in vitro, on the other three histone types, neutralizing their charge and weakening their DNA binding [118,119], or on non-histone targets. Both genes have high sequence similarity, the proteins exhibiting 63% amino acid sequence homology [118,120] and have partially overlapping regulatory functions, functioning as co-activators of HIF1A–*Hypoxia-inducible factor 1 alpha*, which stimulates angiogenesis and tumor growth via the HIF-1–VEGF–TGFB signaling pathway. Inhibition of CBP/CREBBP and EP300 activity in vitro and in vivo blocks tumor growth in neuroblastoma, pancreatic cancer, and acute myeloid leukemia [121]. Through chromatin remodeling, the *EP300* gene product plays a vital role in cell proliferation and differentiation, including hematopoietic stem cells, cell cycle regulation, apoptosis, and DNA damage response. EP300 gene abnormalities are associated with Rubinstein–Taybi syndrome, characterized by a predisposition to malignancies from childhood; epithelial cancers [118,120], such as high-risk pediatric neuroblastomas, in which the EP300 protein is currently required for H3Lys27ac acetylation [122]; esophageal squamous carcinoma, which, in more than 10% of cases, the *EP300* gene is mutated in numerous ways (37 mutations, of which almost 50% are missense), a situation correlated with poor prognosis [123]; or in a very small number of appendiceal cancers, without specifying their type or without details of their prognosis. The CBP/CREBBP protein is critically involved in embryonic development, growth control, and homeostasis [121], with CBP/CREBBP gene mutations causing Rubinstein–Taybi syndrome, while chromosomal translocations affecting it are associated with acute myeloid leukemia [119]. Unlike EP300, in pediatric neuroblastomas, CBP/CREBBP plays a limited role [122] but stimulates lung tumorigenesis via MAPK and CPSF4 signaling pathways, reducing survival and disease-free survival. In appendiceal cancers, mutations in the CBP/CREBBP gene are rarely identified in *low-grade appendiceal mucinous neoplasms*, with no known biological effect [121].

### 4.3. KDR/VEGFR2 Gene

*KDR*/*VEGFR2* gene (4q12) encodes KDR/VEGFR2–*Kinase insert domain receptor*/*Vascular endothelial growth factor receptor 2 protein* (1356 amino acids; 151,527 Da), which functions as a surface receptor for VEGFC, VEGFD, and especially VEGFA proteins, sustaining angiogenic (endothelial proliferation, survival, migration, tubular morphogenesis, and blood vessel sprouting) and tumor (tumor growth, invasion, and therapeutic resistance) processes via HIF-1–VEGF–TGFB, PLCγ–PKC–MEK–MAPK, and PI3K/AKT signaling pathways [124,125]. Overexpression of *KDR*/*VEGFR2* is reported in breast/chest wall angiosarcomas through activating mutations affecting amino acids 717 (Asp717Val) and 1065 (Ala1065Thr) [126] in colorectal cancer, correlating with vascularization and increased metastatic potential of tumors [124,127], with the 889C>T mutation affecting the efficacy of the chemotherapeutic agent bevacizumab [127], in non-small-cell lung cancer [128,129], being correlated with poor clinical outcomes, and, in the case of activation due to copy number gains, with in vitro resistance to platinum-compounds used in chemotherapy, with aggressive phenotypes and with activation of signaling pathways promoting tumor invasiveness [128], and in oropharyngeal neoplasms (own data, unpublished). In appendiceal cancers, a missense mutation p. Gln472His in the *KDR* gene is reported in *mucinous adenocarcinomas of the appendix* without knowing its nature. On the other hand, some mutations in the *KDR* gene seem to be correlated with better responses in non-small-cell lung cancer patients undergoing immunotherapy with pan-cancer for immune checkpoint inhibitors [130].

### 4.4. NOTCH Gene Family

The *NOTCH* gene family comprises four members, encoding the type I transmembrane receptor NOTCH1–4 (*Translocation-Associated Notch Protein TAN-1–4*), which initiates the NOTCH pro-angiogenic signaling pathway (NOTCH–HER/ERBB2–HES/HEY–VEGFR3). Although *NOTCH* genes are located on different chromosomes (NOTCH1 on chromosome 1, NOTCH2 on chromosome 9, NOTCH3 on chromosome 19, and NOTCH4 on chromosome 6), the proteins encoded by them share common structural features, including the extracellular sequence with several epidermal growth factor (EGF)-like repeats, to which three Delta-like proteins (DLL1, DLL2, and DLL4) and two Jagged proteins (JAG1 and JAG2) bind, and the intracellular sequence with several different domains, allowing the binding of different targets. The NOTCH signaling pathway is highly conserved throughout evolution, regulates intercellular interactions, and is involved in processes related to cell fate specification, differentiation, proliferation, angiogenesis, and survival [131]. Depending on the context, NOTCH proteins can stimulate or inhibit tumor development [132]. In appendiceal cancers, mutations in *NOTCH1*, *NOTCH3*, and *NOTCH4* genes are reported [133]. *NOTCH1* gene (9q34.3) encodes the NOTCH1 protein (2555 amino acids; 272,505 Da), which, through the attachment of the JAG1 ligand, participates in upper airway organogenesis, nephron development, stimulation of cell proliferation in the intestinal crypts, and proliferation of glandular epithelium in the endometrium. In an additive manner with NOTCH3, NOTCH1 is involved in the regulation of endocrine cell size and number. Mutations in the *NOTCH1* gene are associated with aortic valve disease, Adams–Oliver syndrome, T-cell acute lymphoblastic leukemia, chronic lymphocytic leukemia, and head and neck squamous cell carcinoma [132]. In lung cancers, the NOTCH1 protein plays a dual role as a suppressor of tumorigenesis in adenocarcinomas, where its overexpression is correlated with favorable progression, and as a stimulator of tumorigenesis in lung squamous cell carcinoma [132]. In appendiceal cancers, *NOTCH1* gene mutations are identified in some cases of *appendiceal goblet cell adenocarcinoma* [10], *mucinous adenocarcinomas of the appendix*, and *low-grade appendiceal mucinous neoplasms*, and in some cases of appendiceal cancers, without further specification [15]. The *NOTCH3* gene (19p13.12) encodes the NOTCH3 protein (2321 amino acids; 243,631 Da), which is expressed in fetal lung mesenchyme, endothelial, and other cell types, stimulating upper airway organogenesis, endometrial luminal epithelial proliferation during the proliferative phase, and mammary gland development. NOTCH3 is a stimulator of tumorigenesis in lung adenocarcinomas and lung squamous cell carcinoma; its overexpression is associated with resistance to chemotherapeutic treatment and reduced survival. On the other hand, regarding cell adhesion, epithelial–mesenchymal transition, and motility, NOTCH3 is a tumor suppressor in small-cell lung cancer and a tumor promoter in non-small-cell lung cancer [132,133]. Diseases associated with abnormalities in the *NOTCH3* gene include cerebral arteriopathy, autosomal dominant, subcortical infarcts and leukoencephalopathy type 1, and lateral meningocele syndrome. Overexpression of the *NOTCH3* gene, mainly due to mutations, is associated with activation of tumor stem cells, leading to tumor growth, increased resistance to chemotherapy, lymph node metastasis, poor prognosis, reduced relapse-free survival and poor overall survival in bladder, colorectal, gastric, head and neck, kidney, liver, lung, ovarian, appendix, and pancreatic cancers, and in leukemia, whereas decreased/blocked expression correlates with reduced proliferation, inhibition of tumor growth, and better disease-free survival in colorectal cancer and reduced proliferation and tumor growth in bladder cancer [134]. The *NOTCH4* gene (6p21.32) encodes the NOTCH4 protein (2003 amino acids; 209,622 Da), expressed predominantly in endothelial cells and stimulating their proliferation; vascular, renal, and hepatic development; and angiogenesis. In the embryonic period, abnormalities in *NOTCH4* gene function due to mutations are associated with vascular defects and abnormal development of several organs, whereas in adulthood, they are identified in schizophrenia, hemangiomas, and breast cancers [135,136], and repeated allergen exposure leads to overexpression of NOTCH4, which contributes to asthma triggering. In cancer, NOTCH4 activity is ambiguous, being a favorable marker in some cancers and associated with poor prognosis in others [136]. Thus, NOTCH4 underexpression increases disease-free survival in long squamous cell carcinoma, and mutations in the NOTCH4 gene appear to lead to improved survival rates in non-small-cell lung cancer [137]. In appendix cancers, mutations of the *NOTCH4* gene are identified in some *low-grade appendiceal mucinous neoplasms*.

### 4.5. FLT1/VEGFR1 Gene

The *FLT1*/*VEGFR1* gene (13q12.3) encodes the FLT1/VEGFR1–*Fms Related Receptor Tyrosine Kinase 1* protein (1338 amino acids; 150,769 Da), which functions as a receptor for VEGF1/VEGFA and VEGFB, some of the most potential factors favorably involved in angiogenesis, and PGF–*placental growth factor*. FLT1/VEGFR1 is part of the HIF1–VEGF signaling pathway, playing an essential role in the development of embryonic vasculature in limiting the excessive proliferation of embryonic endothelial cells, and in adult PGF-mediated endothelial proliferation, the regulation of angiogenesis, cell survival, cell migration, macrophage function, chemotaxis, and cancer cell invasion. By forming heterodimers with KDR, FLT1/VEGFR1 modulates its function. By excessively binding VEGF1/VEGFA, FLT1/VEGFR1 reduces its availability to the KDR receptor, limiting signal transmission through the VEGF pathway since FLT1/VEGFR1 can activate a large number of signaling pathways. Thus, by activating PLGC, FLT1/VEGFR1 induces activation of protein kinase C and signal transmission through the RAF–MEK–ERK pathway. On the other hand, FLT1/VEGFR1 mediates PIK3R1 phosphorylation and MAPK1/ERK2 and MAPK3/ERK1 activation, contributing to the stimulation of PIK3/AKT and MAPK signaling pathways. FLT1/VEGFR1-associated diseases are pre-eclampsia and eclampsia [138]. The FLT1/VEGFR1 gene is overexpressed in colorectal cancers and is associated with poor prognosis [124,139], whereas in a minimal number of appendiceal cancers, it carries mutations, the nature of which is not specified.

## 5. Mutations in Genes Whose Proteins Are Involved in Acquiring Insensitivity to Anti-Growth Signals

Acquiring resistance to anti-growth signals through the TGFB–TGFBR–SMAD signaling pathway is a critical step in tumor growth, allowing tumor cells to override the proliferation-inhibitory signals that ensure normal homeostasis. The TGFB–TGFBR–SMAD signaling pathway initiators, TGFB1–*Transforming Growth Factor β1*, TGFB2–*Transforming Growth Factor β2*, and TGFB3–*Transforming Growth Factor β3*, bind to the TGFBR2 receptor, which then recruits and phosphorylates TGFBR1. Activated by phosphorylation, TGFBR1 phosphorylates SMAD2–*Mothers Against Decapentaplegic Homolog 2* and SMAD3–*Mothers Against Decapentaplegic Homolog 3*, which further recruits SMAD4–*Mothers Against Decapentaplegic Homolog 4*, and, after translocation to the nucleus, regulate transcription of TGFB target genes involved in numerous biological processes, including the regulation of proliferation, migration, differentiation, immune response, apoptosis, and G1-phase cell arrest. In non-transformed cells, TGFB–TGFBR–SMAD signaling inhibits tumor growth and proliferation, but in malignancies, its role becomes the opposite, stimulating neoplastic processes [140]. In appendiceal cancers, mutations in *TGFBR* and *SMAD* genes are reported.

### 5.1. TGFBR Gene Family

**Figure 8:**
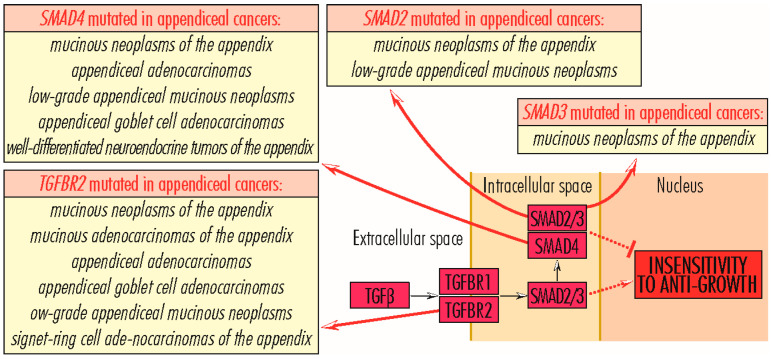
TGFB signaling pathway in cancer, pointing out the appendiceal cancer types in which *TGFBR2*, *SMAD2*, *SMAD3*, and *SMAD4* genes are mutated (adapted from KEGG Pathways, 2020 [14]).

The TGFBR gene family encodes three proteins, TGFBR1, TGFBR2, and TGFBR3, which are receptors for signals transmitted by TGFB proteins. In the TGFB–TGFBR–SMAD signaling pathway, TGFB proteins bind to TGFBR2–*Transforming Growth Factor Beta Receptor 2*, which, when activated, binds TGFBR1–*Transforming Growth Factor Beta Receptor 1*, but the reverse process is not possible. The *TGFBR2* gene (3p24.1) encodes the TGFBR2 protein (567 amino acids; 64,568 Da), which auto-phosphorylates or recruits and phosphorylates other downstream proteins, including TGFBR1, involved in the regulation of cell proliferation, G1-phase cell cycle arrest, wound healing, immunosuppression, and tumorigenesis. Deficiencies due to mutations in *TGFBR2* gene function are associated with Marfan syndrome, Loeys–Deitz aortic aneurysm syndrome, and tumor progression. In cancers, mutations in the *TGFBR2* gene are identified in a small number of cases (less than 5%) in lung adenocarcinoma, lung squamous cell carcinoma, esophageal cancer, and head and neck squamous cancer [7]. Some mutations, such as the one leading to the Glu526Gln alteration, lead to loss of kinase activity and signaling functions, since others, including Arg537Pro, amplify kinase activity, with loss of SMAD2/SMAD3 activation and constitutive SMAD1/SMAD5 activation. These mutations are common in carcinoma cells, conferring high mobility and invasiveness [14,141]. Some mutations identified in non-small-cell lung carcinoma confer resistance to treatment with immune checkpoint inhibitors [7,14]. Mutations in the *TGFBR2* gene are reported in a small number of cases of *mucinous neoplasms of the appendix*, with possible loss of function [7], and in less than 10% of cases of *mucinous adenocarcinomas of the appendix*, *appendiceal adenocarcinomas*, *appendiceal goblet cell adenocarcinomas*, *low-grade appendiceal mucinous neoplasms*, and *signet ring cell adenocarcinomas of the appendix*. *TGFBR1* gene (9q22.33) encodes the TGFBR1 protein (503 amino acids; 55,960 Da). It is phosphorylated by TGFB-activated TGFBR2 and downstream phosphorylates SMAD2 and SMAD3 proteins from the TGFB–TGFBR–SMAD signaling pathway, or TRAF6, involved in activation of the PI3K/AKT and JNK signaling pathways. In these pathways, TGFBR1 is involved in G-phase cell cycle arrest in epithelial and hematopoietic cells, the regulation of cellular apoptosis, epithelial–mesenchymal transition, mesenchymal cell proliferation and differentiation, extracellular matrix synthesis, wound healing, immunosuppression, cancer cell invasion, and tumorigenesis. Defects in *TGFBR1* gene function are associated with Loeys–Dietz aortic aneurysm syndrome, multiple self-healing squamous epitheliomas [140,141,142], and the development of some cancers. Thus, TGFBR1*6A polymorphism, consisting of nine-base pair in-frame deletion within exon 1, and IVS7+24G>A polymorphism and TGFBR1 haploinsufficiency reduce TGFBR1 protein functionality, are correlated with tumor cell proliferation and early onset adenocarcinoma, and increase the risk of colon, colorectal, breast, and osteosarcoma cancers [143,144,145,146]. In appendiceal tumors, mutations in the *TGFBR1* gene are identified in a few cases of *mucinous neoplasms* with possible loss of function [147] (Figure 8).

### 5.2. SMAD Gene Family

The *SMAD* gene family comprises eight related genes, *SMAD1*–*SMAD8*, encoding *SMAD-Mothers Against Decapentaplegic Homologues*, which transmit signals from TGFBR1 to the nucleus. *SMAD* genes are located in four chromosomes (4, 13, 15, and 18), and three of them, *SMAD2*, *SMAD4*, and *SMAD7*, are clustered in the 18q21.1 region, frequently deleted in cancers; three of them, *SMAD3*, *SMAD6*, and *SMAD5*, are clustered in chromosome 15 (*SMAD3* and *SMAD6* in region 15q21–22, and *SMAD5* in region 15q31); and *SMAD1* and *SMAD8* are located in chromosomes 4 and 13. SMAD2 and SMAD3 are regulated by TGFβ/activin; SMAD1, SMAD5, and SMAD8 are activated by BMP receptors; SMAD4 is commonly recruited by SMAD2/SMAD3; and SMAD6 and SMAD7 have inhibitory activity on the TGFB–TGFBR–SMAD signaling pathway. In the absence of SMAD4, SMAD2 and SMAD3 may regulate specific transcriptional pathways of oncogenic signals, probably through interaction with IKK–*Inhibitor of Nuclear Factor Kappa B Kinase* or TIF1G–*Transcriptional Intermediary Factor 1γ*. *SMAD* genes are essential for embryogenesis, expressed throughout embryonic development, with differences in their action, but to a somewhat lesser extent also in adult tissues. Thus, in the embryo, *SMAD6* and *SMAD7* are highly expressed in the developing cardiovascular system and sporadically expressed in other tissues, such as bone and testis. The *SMAD4* gene is also highly expressed during embryonic development, especially in the epithelial crypts of the small intestine. The other *SMAD*s (*SMAD1*, *SMAD5*, *SMAD8*, and *SMAD2*/*SMAD3*) are expressed in all tissue types. Their absence during embryonic development is lethal (especially *SMAD2*, *SMAD4*, or *SMAD5*) or leads to defects in gastrulation, mesoderm induction, and endoderm formation; numerous cardiovascular, immune, digestive, and joint abnormalities; as well as metastatic colon cancer or other intestinal tumors [14,148,149]. Mutations in the *SMAD2*, *SMAD3*, and *SMAD4* genes are associated with cancers of the intestine, pancreas, or appendix. The *SMAD2* gene (18q21.1) encodes the SMAD2–*SMAD Mothers Against Decapentaplegic Homolog 2* protein (467 amino acids; 52,306 Da). Activin type 1 or TGFBR1 phosphorylates it, subsequently recruits SMAD4 and transmits signals to the nucleus, and is thought to suppress tumor development. Mutations of the *SMAD2* gene are rare, leading to congenital heart defects with or without heterotaxy and Loeys–Dietz syndrome 6. In cancers, the frequency of mutations is also low and found in cervical, colorectal, hepatocellular carcinoma, breast, non-small-cell lung, and appendiceal cancers [150,151], including a small number of cases of *mucinous neoplasms of the appendix*, with possible loss of function, and *low-grade appendiceal mucinous neoplasms* [148]. The *SMAD3* gene (15q22.33) encodes the SMAD3–*SMAD Mothers Against Decapentaplegic Homolog 3* protein (425 amino acids; 48, 081 Da), which functions analogously to SMAD2, as a transcription factor and tumor suppressor, through several mechanisms, including apoptosis [152]. Mutations in the *SMAD3* gene can disrupt the TGFB–TGFBR–SMAD signaling pathway and are associated with aneurysms–osteoarthritis syndrome and Loeys–Dietz syndrome 3. In cancers, *SMAD3* gene mutations are rare and are identified in colorectal adenocarcinomas, choriocarcinomas, and rarely in pancreatic ductal adenocarcinomas and appendiceal cancers [150,153], including *mucinous neoplasms of the appendix*, with possible loss of function [7]. The *SMAD4* gene (18q21.2) encodes the common SMAD4–*SMAD Mothers Against Decapentaplegic Homolog 2* protein (552 amino acids; 60,439 Da), recruited by SMAD2/SMAD3, with which it forms a heterotrimeric complex in the nucleus, through which biological signals from the TGFB–TGFBR–SMAD signaling pathway are transmitted. SMAD4 has tumor suppressor activity, and, in its absence, the SMAD2/SMAD3 heterodimer mediates the oncogenic effects of TGFB by activating a SMAD4-independent signaling pathway. SMAD4-associated diseases include Myhre syndrome, juvenile polyposis syndrome, and hereditary hemorrhagic telangiectasia syndrome. Mutations in the *SMAD4* gene are present in T lymphocytes and frequently in pancreatic ductal adenocarcinomas (in 50% of cases), in which they are associated with metastasis and poor overall survival [148,154]. Rarely, mutations or deletions of the *SMAD4* gene occur in other cancers, such as colorectal cancer [150]; head and neck cancer, in which it is associated with metastasis and in cancers of the appendix, including *mucinous neoplasms of the appendix*, with possible loss of function [7]; *appendiceal adenocarcinomas* [15,16,30,33], in which their frequency exceeds 10%; and *low-grade appendiceal mucinous neoplasms*, *appendiceal goblet cell adenocarcinomas*, and *well-differentiated neuroendocrine tumors of the appendix* [37].

## 6. Mutations in Genes Whose Proteins Are Involved in the Organization of the Extracellular Matrix

Extracellular matrices (ECMs) are multiplicate well-organized three-dimensional architectural networks with critical structural and functional roles in tissue organization and remodeling and the regulation of cellular processes [155,156]. The building blocks of these ultrastructures are collagens, proteoglycans and glycosaminoglycans, elastin and elastic fibers, laminins, fibronectin, and other proteins/glycoproteins such as matricellular proteins [157]. Cells sense the biochemical and mechanical properties of the ECM through specialized transmembrane receptors that include integrins, discoidin domain receptors (DDRs), and syndecans. Once activated, these ECM receptors recognize specific motifs within the ECM molecule that induce conformational changes within the receptor that promote molecular associations between the receptor and intracellular adhesion plaque proteins that activate signaling to influence cell behavior. Diseases such as cancer, defined by disrupted tissue homeostasis and loss of a differentiated phenotype, are accompanied by alterations in the composition, organization, and mechanical properties of the ECM that contribute not only to malignant transformation but also to tumor progression and metastasis [158].

The development of invasive tumors and their subsequent spread to other body parts involve distinct biological changes in the extracellular matrix (ECM). Various steps can be observed during this process. To become invasive, cancer cells must modify the basement membrane by thinning it and reducing the levels of an important protein called laminin-111 [158]. Additionally, around precancerous lesions, there is an increase in the amount and thickness of interstitial collagen [159]. Once tumor cells have invaded the surrounding tissue, their ability to spread is facilitated by a remodeled interstitial ECM. These ECM changes, such as increased stiffness and reorganization into linear bundles of type I collagen, are often accompanied by fibronectin [160]. These altered ECM characteristics create pathways that promote the directed migration of tumor cells toward blood vessels, allowing them to enter the bloodstream efficiently [161]. Importantly, the presence of perpendicular, linearized, and stiffened collagen bundles has been linked to poorer prognosis in breast cancer patients [162]. Moreover, aggressive types of breast cancer, such as HER2+ and triple-negative, exhibit a stiffer invasive stroma with higher levels of linearized collagens [159].

The associations between the tumor ECM and the metastatic cascade not only provide evidence of a clinical connection between ECM remodeling and the development of malignancy and metastasis but also suggest that there is likely a cause-and-effect relationship. This knowledge could be utilized to develop predictive biomarkers or targeted therapeutics to reduce patient mortality. Among the genes involved in extracellular matrix organization, in appendiceal tumors, only *COL5A3*, *COL6A3*, and *ZNF469* seem to be mutated to a small extent.

The gene products of *COL5A3* (19p13.2) and *COL6A3* (2q37.3) genes, COL5A3–*Collagen Type V Alpha 3 Chain* (1745 amino acids; 172,121 Da) and COL6A3–*Collagen Type VI Alpha 3 Chain* (3177 amino acids; 343,669 Da) proteins, are involved in the organization of the extracellular matrix and thus in cell adhesion. Diseases associated with defects in the COL5A3 gene are Ehlers–Danlos syndrome and colon small cell carcinoma and those associated with abnormalities in the COL6A3 gene, Dystonia 27 and Ullrich congenital muscular dystrophy 1, whereas mutations in these genes are present in a minimal extent in some *mucinous neoplasms of the appendix* [7,163,164].

The *ZNF469* gene (16q24.2) encodes the ZNF469–*Zinc Finger Protein 469* (3925 amino acids; 410,202 Da), whose low-percent homology with some collagens suggests its involvement in transcription or regulation of synthesis and organization of collagen fibers. Among the diseases associated with ZNF469 are brittle cornea syndrome 1 and keratoconus 1 [165]. In addition, mutations in this gene are reported in some *mucinous neoplasms of the appendix* cases [7].

## 7. Other Genes Showing Mutations in Appendix Cancer

Included in this category are genes whose products are not involved, according to KEGG Pathways, 2020, in critical signaling pathways in cancer, i.e., RAS–RAF–MEK–ERK, PI3K–PKB/AKT, WNT, JAK–STAT, PLCɣ–PKC–MEK–MAPK, ESR/JUP–FOS/JUN/SP1/NCOA/ESR, MYC/c-MYC–CCND–RB1–E2F, RAS–RALGDS–RHO–JNK, NOTCH–HER/ERBB2–HES/HEY–VEGFR3, HIF1–VEGF, and TGFB–TGFBR–SMAD, but this does not reduce their importance in the tumor process. A brief review of these genes will be given below, some being included in certain functional categories (DNA metabolism/expression, cell junctions/adhesion, enzymes) and others having various functions and being included in a separate category.

### 7.1. Genes Involved in DNA Metabolism/Expression

The *ATRX* gene (Xq21.1) encodes the *ATRX Chromatin Remodeler* (2492 amino acids; 282,587 Da), which contains an ATPase/helicase domain, is a member of the SWI/SNF family and is involved in embryonic patterning, cell lineage gene regulation, cell cycle control, and the transcription of specific genes by altering chromatin structure. Among the diseases produced by alterations in the typical structure of the ATRX gene are alpha-thalassemia myelodysplasia syndrome and intellectual disability–hypotonic facies syndrome, X-linked, 1 [166], and its mutations are also present in some appendiceal cancers [16].

The *BRCA1* gene (17q21.31), which encodes the *BRCA1 DNA Repair-Associated* protein (1863 amino acids; 207,721 Da), and the *BRCA2* gene (13q13.1), which encodes the *BRCA2 DNA Repair-Associated* protein (3418 amino acids; 384,230 Da), contribute to maintaining genome stability [167,168]. Defects in these genes lead to Fanconi anemia and complementation group D1, and mutations are present in breast cancer, *appendiceal adenocarcinomas*, *mucinous adenocarcinomas of the appendix*, and *signet ring cell adenocarcinoma* [8].

The *FANCA* gene (16q24.3) encodes the FANCA–*Fanconi Anemia Complementation Group A* protein (1455 amino acids; 162,775 Da), a member of the FANC group, which may be involved in DNA repairing processes, interstrand DNA cross-link repair, and maintenance of chromosome stability. Defects in the FANCA gene are identified in Fanconi anemia, complementation group a, and pituitary stalk interruption syndrome [169]. However, this gene is rarely mutated in appendix cancers, as in a single case of *low-grade appendiceal mucinous neoplasm* [32].

The *KDM6A* gene (Xp11.3) encodes the KDM6A–*Lysine Demethylase 6A* protein (1401 amino acids; 154,177 Da), which catalyzes the demethylation of tri/di-methylated histone H3. Defects in the functioning of the *KDM6A* gene are present in Kabuki syndromes 1 and 2 [170], and mutations in this gene are reported in a few cases of *appendiceal goblet cell adenocarcinomas* [32].

The *KMT2D/MLL2* gene (12q13.12) encodes the KMT2D/MLL2–*Lysine Methyltransferase 2D* protein (5537 amino acids; 593,389 Da), which methylates the Lys-4 position of histone H3 and coactivates the estrogen receptor. When mutated, it is associated with Kabuki syndrome 1 and choanal atresia–athelia–hypothyroidism–delayed puberty–short stature syndrome [171]. However, in appendiceal cancers, mutations in the *KTM2D* gene are rarely mutated, with two cases of *appendiceal goblet cell adenocarcinomas* reported so far [32].

The *RAD51C* gene (17q22) encodes the RAD51C–*RAD51 Paralog C* protein (376 amino acids; 42,190 Da), a member of the RAD51 family, involved in the homologous recombination and repair of DNA. The 17q22 region is frequently amplified in breast cancers, suggesting that amplifying the *RAD51C* gene may affect tumor progression. Defects in this gene are present in familial breast–ovarian cancer and Fanconi anemia, complementation group O [172]. In appendiceal cancers, the *RAD51C* gene is reported with the pathogenic mutation c.955C>T (Arg319*) in a single case of *low-grade appendiceal mucinous neoplasm* [32]. 

The *SETD2* gene (3p21.31) encodes the SETD2–*SET Domain Containing 2, Histone Lysine Methyltransferase* (2564 amino acids; 287,597 Da), involved in chromatin structure modulation during elongation, regulation of DNA mismatch repair in G1 and early S phase, and DNA double-strand break repair in response to DNA damage, through the formation of H3K36me3 and endoderm development. It also mediates protein methylation and is a tumor suppressor [173]. However, its defects are found in some diseases, including Luscan–Lumish and Sotos Syndromes and appendiceal cancers [32].

The *TRPS1* gene (8q23.3) encodes the TRPS1–*Transcriptional Repressor GATA Binding 1* protein (1281 amino acids; 141,521 Da), which represses GATA-regulated genes and binds to a dynein light chain protein, regulates chondrocyte proliferation and differentiation, and is involved in vertebrate development. Defects in the *TRPS1* gene are identified in tricho-rhino-phalangeal syndrome types I–III [174]. In addition, in appendiceal cancers, the *TRPS1* gene is mutated in *mucinous neoplasms of the appendix* [7].

### 7.2. Genes Involved in Cell Junctions/Adhesion

The *CDH1* gene (16q22.1) encodes the CDH1–*Cadherin 1* (882 amino acids; 97,456 Da), which acts as a calcium-dependent adhesion protein. Deficiencies in *CDH1* gene function lead to blepharocheilodontic syndrome 1 and diffuse gastric and lobular breast cancer syndrome. Loss of *CDH1* gene function leads to gastric, breast, colorectal, thyroid, and ovarian cancer [175], and its mutations have been reported in *appendiceal goblet cell adenocarcinomas* [27] and, very rarely, in *mucinous adenocarcinomas of the appendix*.

The *CTNNA1* gene (5q31.2), which encodes CTNNA1–*Catenin Alpha 1* (906 amino acids; 100,071 Da), and *CTNNB1* gene (3p22.1), which encodes CTNNB1–*Catenin Beta 1* (781 amino acids; 85,497 Da), are part of the catenin gene family, whose products are involved in the formation of adherens junctions. Defects in the *CTNNA1* gene are identified in macular dystrophy, patterned, 2, and butterfly-shaped pigment dystrophy, since an abnormal *CTNNB1* gene is detected in pilomatrixoma and colorectal cancer [176,177]. Furthermore, in appendiceal cancers, mutations in the *CTNNA1* gene are occasionally found in *appendiceal goblet cell adenocarcinomas*, and mutations in *CTNNB1* are found in *mucinous neoplasms of the appendix* [7], *low and high-grade appendiceal mucinous neoplasms*, and *appendiceal goblet cell adenocarcinomas* [10,178].

The *CNTNAP2* gene (7q35-q36.1) encodes the CNTNAP2–*Contactin-Associated Protein 2* (1331 amino acids; 148,167 Da), a member of the neurexin family, involved in nervous cell adhesion. CNTNAP2 defects are present in Pitt–Hopkins-like syndrome 1 and autism 15 [179] since its mutations are detected in some *mucinous neoplasms of the appendix* [7].

The *LAMA1* gene (18p11.31) encodes the LAMA1–*Laminin Subunit Alpha 1* protein (3075 amino acids; 337,084 Da), part of the laminin gene family. Laminins are heterotrimeric proteins consisting of alpha, beta, and gamma subunits, making the significant component of basement membranes and being involved in cell adhesion, differentiation, migration, signaling, neurite outgrowth, and metastasis. Diseases associated with LAMA1 defects are Poretti–Boltshauser syndrome and myopia [180]. In addition, in appendix cancers, mutations in its structure are identified in a few cases of *mucinous neoplasms of the appendix* [7].

The *SPTA1* gene (1q23.1) encodes the SPTA1–*Spectrin Alpha, Erythrocytic 1* protein (2419 amino acids; 280,014 Da), which binds the plasma membrane to the cytoskeleton, in cell shaping, arrangement of transmembrane proteins, and organization of organelles. Disruptions in *SPTA1* gene function due to mutations lead to red blood cell disorders, such as elliptocytosis-2, pyropoikilocytosis, and spherocytosis type 3 [181]. In addition, in appendiceal cancers, mutations in this gene are reported in *mucinous neoplasms of the appendix* [30].

### 7.3. Genes Encoding for Enzymes

The *ALK* gene (2p23.2-p23.1) encodes the *ALK Receptor Tyrosine Kinase* (1620 amino acids; 176,442 Da), which is part of the insulin receptor superfamily and receptor tyrosine kinases (RTKs), with an essential role in the development of the brain and specific neurons in the nervous system and whose abnormalities are present in neuroblastoma 3 and neuroblastoma. Mutations of the *ALK* gene are also reported in appendiceal cancers without their nature being specified [182].

The *ARID1A* gene (1p36.11), which encodes the ARID1A–*AT-Rich Interaction Domain 1A* (2285 amino acids; 242,045 Da), and the *ARID2* gene (12q12), which encodes the ARID2–*AT-Rich Interaction Domain 2* (1835 amino acids; 197,391 Da), are members of the SWI/SNF family of helicases/ATPases, which act as regulators of embryonic patterning, cell lineage gene regulation, cell cycle control, and transcription of specific genes by altering chromatin structure. Among the diseases associated with defects in this gene are Coffin–Siris syndrome 1 and Coffin–Siris syndrome 2 [183]. Mutations in *ARID1A* and *ARID2* genes are present in appendiceal cancers in general [7], with *ARID1A* gene mutations reported in *appendiceal goblet cell adenocarcinomas* [11], *appendiceal adenocarcinomas*, *low-grade appendiceal mucinous neoplasms*, *signet ring cell adenocarcinoma*, and *mucinous adenocarcinomas of the appendix*, and mutations in the *ARID2* gene in *appendiceal goblet cell adenocarcinomas* and *appendiceal adenocarcinomas* [11,184]. 

The *ATM* gene (11q22.3) encodes the *ATM Serine/Threonine Kinase* (3056 amino acids; 350,687 Da), which belongs to PI3/PI4-kinase family and is involved in regulating the functions of a large number of proteins, including p53, BRCA1, CHK2, RAD17, RAD9, and NBS1. Among the diseases produced by defects in this gene are ataxia-telangiectasia and mantle cell lymphoma [185]. *ATM* gene mutations are present in *appendiceal goblet cell adenocarcinomas* [27], *mucinous adenocarcinomas of the appendix*, *low-* and *high-grade appendiceal mucinous neoplasms* [15,16], and *well-differentiated neuroendocrine tumors of the appendix* [37].

The *CARD11* gene (7p22.2) encodes CARD11–*Caspase Recruitment Domain Family Member 11* (1154 amino acids; 133,284 Da), which is part of the membrane-associated guanylate kinase (MAGUK) family, with roles in the assembly of protein complexes in specialized regions of the plasma membrane, and the CARD family of proteins, which carry characteristic CARD caspase-associated recruitment domains. These domains interact with the BCL10 Immune Signaling Adaptor, which positively regulates apoptosis and NF-kappaB activation. Abnormalities in *CARD11* gene function are found in B-cell expansion with NfkB and T-cell anergy and immunodeficiency 11 and in appendiceal cancers [8,186]. 

The *DCLK1* gene (13q13.3) encodes the DCLK1–*Doublecortin Like Kinase 1* (740 amino acids; 82,224 Da), a member of both the protein kinase superfamily and the doublecortin family, being involved in neurogenesis, neuronal apoptosis, microtubule polymerization, multiple protein–protein interactions, neuronal migration, and retrograde transport. Its defects are present in band heterotopia and Zellweger syndrome [187]. In addition, in some *mucinous neoplasms of the appendix* cases, there are reported mutations in the *DCLK1* gene [7].

The *DIS3* gene (13q21.33) encodes the *DIS3 Homolog, Exosome Endoribonuclease, and 3′*-*5′ Exoribonuclease* (958 amino acids; 109,003 Da), which is part of the nuclear exosome RNAase complex in the nuclear membrane and cytosol and, by regulating the activity of 3′-5′-exoribonuclease and guanyl-nucleotide exchange factor, is involved in CUT and rRNA catabolism. Therefore, its structure and function defects lead to plasma cell neoplasm and myeloma. At the same time, in appendiceal cancers, mutations in the *DIS3* gene have been reported in a single case of *low-grade appendiceal mucinous neoplasm* [10,188].

The *FH* gene (1q43) encodes the FH–*Fumarate Hydratase* protein (510 amino acids; 54,637 Da), which catalyzes the conversion of fumarate to L-malate and promotes energy production in the form of NADH. Deficiencies in the functioning of this gene are present in fumarase deficiency and hereditary leiomyomatosis and renal cell cancer [189]. In appendiceal cancers, the *FH* gene appears mutated in a single case of *low-grade appendiceal mucinous neoplasm* [32].

The *IDH2* gene (15q26.1) encodes the IDH2–*Isocitrate Dehydrogenase (NADP(+)) 2* protein (452 amino acids; 50,909 Da), a member of the IDH family, whose members catalyze the oxidative decarboxylation of isocitrate to 2-oxoglutarate. Defects in the *IDH2* gene are associated with d-2-hydroxyglutaric aciduria 2 and multiple enchondromatosis, Maffucci type. At the same time, mutations in it are reported in some cancers, such as sarcomas, hematologic malignancies, colon cancer, and brain cancer [190], also reported in a single case of *mucinous neoplasm of the appendix*.

The *PRKACA* gene (19p13.12) encodes the PRKACA–*Protein Kinase CAMP-Activated Catalytic Subunit Alpha* (351 amino acids; 40,590 Da), which is part of the PKA-protein kinase A structure, the latter phosphorylating, in a cAMP-dependent manner, several proteins involved in differentiation, proliferation, and apoptosis. Defects in the *PRKACA* gene are present in several hyperplasias and adenomas of the adrenal cortex and in ACTH-independent Cushing’s syndrome, cardioacrofacial dysplasia 1, and primary pigmented nodular adrenocortical disease 4 [191]. In addition, mutations in the *PRKACA* gene are found in a few cases of *mucinous neoplasms of the appendix* [7].

The *PTPN11* gene (12q24.13) encodes the PTPN11–*Protein Tyrosine Phosphatase Non-Receptor Type 11* protein (593 amino acids; 68,011 Da), a member of the protein tyrosine phosphatase (PTP) family, which are signaling molecules in pathways including cell growth, differentiation, mitotic cycle, and oncogenic transformation. The *PTPN11* gene is expressed in most tissues and regulates mitogenic activation, metabolic control, transcriptional regulation, and cell migration, and mutations in this gene are found in Noonan syndrome 1, juvenile myelomonocytic leukemia [192], and, less commonly, in some appendicular cancers, including *mucinous neoplasms of the appendix* [15,15,32].

The *RHPN2* gene (19q13.11) encodes the RHPN2–*Rhophilin Rho GTPase Binding Protein 2* (686 amino acids; 76,993 Da), a member of the rhophilin family of Ras-homologous (Rho)-GTPase binding proteins, and probably involved in the organization of the actin cytoskeleton. Defects in *the RHPN2* gene are identified in diseases including hereditary mixed polyposis syndrome and isolated cleft palate [193]. However, in appendiceal cancers, mutations in the *RHPN2* gene are rarely found in *appendiceal goblet cell adenocarcinomas* and *appendiceal adenocarcinomas* [8,10].

The *SMARCA4* gene (19p13.2) encodes SMARCA4–*SWI/SNF-Related, Matrix-Associated, Actin-Dependent Regulator of Chromatin, Subfamily A, Member 4* (1647 amino acids; 184,646 Da), a member of the SWI/SNF family of proteins with helicase and ATPase activities, which likely regulates transcription of some genes and is thus involved in some biological processes. Defects in protein function are identified in some diseases, including Coffin–Siris syndrome 4 and predisposition to rhabdoid tumors syndrome 2 [194]. In appendiceal cancers, the Ala1536Ser variant with uncertain significance is reported in a case of *low-grade appendiceal mucinous neoplasm* [32].

The *TRRAP* gene (7q22.1) encodes the TRRAP–*Transformation/Transcription Domain-Associated Protein* (3859 amino acids; 437,600 Da), a member of the phosphoinositide 3-kinase-related kinases (PIKK) family and a standard component of many histone acetyltransferases (HAT) complexes, being involved in transcription and DNA repair. Defects in *the TRRAP* gene are found in some diseases, including developmental delay with/without dysmorphic facies and autism and autosomal dominant deafness 75, and dysregulations are reported in certain types of cancer, including glioblastoma multiforme [195], *appendiceal adenocarcinomas*, and *appendiceal goblet cell adenocarcinomas* [10].

The *USP9X* gene (Xp11.4) encodes the USP9X–*Ubiquitin Specific Peptidase 9 X-Linked* protein (2554 amino acids; 290,463 Da), a member of the C19 family of peptidases and similar to ubiquitin-specific proteases. It may play an essential role in regulating protein turnover by preventing protein degradation. Mutations in the *USP9X* gene are associated with Turner syndrome, syndromic and X-linked female-restricted intellectual developmental disorder, and X-linked intellectual developmental disorder [196], being also reported in a single case of *appendiceal goblet cell adenocarcinoma* [10].

### 7.4. Genes with Various Cellular Activities

The *ABCA7* gene (19p13.3) encodes the ABCA7–*ATP Binding Cassette Subfamily A Member 7* protein (2146 amino acids; 234,350 Da), which plays a role in the transport of molecules across extra- and intra-cellular membranes, is associated with Alzheimer disease 9 and early-onset, autosomal dominant Alzheimer disease, and is mutated in a minimal number of cases of *mucinous neoplasms of the appendix* [7,197]. 

The *ANKRD24* gene (19p13.3) encodes the ANKRD24–*Ankyrin Repeat Domain 24* protein (1146 amino acids; 124,187 Da), whose abnormalities are identified in Spinocerebellar Ataxia 17. In addition, mutations of this gene are reported in a small number of *mucinous neoplasms of the appendix* [7,198].

The *APOB* gene (2p24.1) encodes the APOB–*Apolipoprotein B* (4563 amino acids; 515,605 Da), a ligand for LDL (low-density lipoproteins), and is the primary apolipoprotein of chylomicrons and low-density lipoproteins (LDL). Its abnormalities are present in hypobetalipoproteinemia, familial, 1, and hypercholesterolemia, familial, 2. In addition, mutations in this gene are detected in a few cases of *mucinous neoplasms of the appendix* [7,199].

The *ASXL1* gene (20q11.21) encodes the ASXL1–*ASXL Transcriptional Regulator 1* (1541 amino acids; 165,432 Da), involved in embryonic development. Defects of this gene are present in Bohring–Opitz and myelodysplastic syndromes, and some mutations are also identified in appendiceal cancers [7,200].

The *BCOR* gene (Xp11.4) encodes the BCOR–*BCL6 Corepressor* (1755 amino acids; 192,189 Da), involved in the germinal center formation and, probably, apoptosis. Defects in the *BCOR* gene are present in syndromic microphthalmia 1 and 2, and mutations are detected in some appendicular cancers [16,201].

The *CRY2* gene (11p11.2) encodes the CRY2–*Cryptochrome Circadian Regulator 2* flavin adenine dinucleotide-binding protein (593 amino acids; 66,947 Da), an essential component of the circadian core oscillator complex, involved in the regulation of the circadian clock. Defects in the *CRY2* gene lead to advanced sleep phase syndrome and severe congenital neutropenia 2, since mutations are found in some *mucinous neoplasms of the appendix* [7,202].

The *DOCK3* gene (3p21.2) encodes the DOCK3–*Dedicator of Cytokinesis 3* guanine nucleotide exchange factor (2030 amino acids; 233,103 Da). It is a member of the DOCK (dedicator of cytokinesis) family. It is expressed predominantly in the central nervous system, stimulating axon growth by stimulating membrane recruitment of the WAVE complex and activating the RAC1 protein. Defects in this gene are associated with neurodevelopmental disorders with impaired intellectual development, hypotonia, and ataxia and hypotonia [203]. In appendiceal cancers, mutations in the *DOCK3* gene occur sparsely in some cases of *mucinous neoplasms of the appendix* [7].

The *DOK6* gene (18q22.2) encodes DOK6–*Docking Protein 6* (331 amino acids; 38,318 Da), a member of the DOK family of intracellular adaptors that activate retinoate. Among the diseases associated with defects in *DOK6* gene function are malignant iris melanoma and Hirschsprung disease 1 [204]. In appendiceal cancers, some mutations occur in a few *mucinous neoplasms* [7].

The *EEF1A1* gene (6q13) encodes the EEF1A1–*Eukaryotic Translation Elongation Factor 1 Alpha 1* (462 amino acids; 50,141 Da) isoform of the alpha subunit of the elongation factor-1 complex. Isoform A1 is expressed in the brain, placenta, lung, liver, kidney, and pancreas, where it is involved in the enzymatic delivery of aminoacyl tRNA to the ribosome. Defects in the *EEF1A1* gene are identified in Felty syndrome and eukaryotic translation elongation factor 1 alpha-1-like 14 [205]. In addition, in appendiceal cancers, the *EEF1A1* gene is sparsely mutated in *mucinous neoplasms of the appendix* [7]. 

The *EPHA10* gene (1p34.3) encodes the EPHA10–*Ephrin Receptor A10* (1008 amino acids; 109,716 Da), a member of the most prominent family of tyrosine kinase receptors, which regulate cell attachment, shape, and mobility of neuronal and epithelial cells. Its defects are associated with hypogonadotropic hypogonadism 6 with or without anosmia [206]. In addition, mutations in the *EPHA10* gene are reported in a few cases of *mucinous neoplasms of the appendix* [7]. 

The *FAT1* gene (4q35.2), which encodes FAT1–*FAT Atypical Cadherin 1* or *Protocadherin Fat 1* (4588 amino acids; 506,273 Da), and the *FAT4* gene (4q28.1), which encodes *FAT Atypical Cadherin 4* or *Protocadherin Fat 4* (4981 amino acids; 542,687 Da), are members of the protocadherin family. The *FAT1* gene is expressed in various fetal epithelia, modulating cellular polarization, cell migration, and cell–cell contact. Defects in the *FAT1* gene are present in Myasthenic Syndrome, Congenital, 12, Nephrotic Syndrome, Type 1, and appendiceal cancers [207,208]. The *FAT4* gene is involved in the regulation of planar cell polarity. Among the diseases associated with defects in the *FAT4* gene are van Maldergem syndrome 2 and Hennekam lymphangiectasia–lymphedema syndrome 2. In appendiceal cancers, mutations in the *FAT4* gene are reported in a small number of cases of *mucinous neoplasms of the appendix* [7] and in a case of *low-grade appendiceal mucinous neoplasms*.

The *FBXW7* gene (4q31.3) encodes the FBXW7–*F-Box and WD Repeat Domain Containing 7* (707 amino acids; 79,663 Da), a member of the F-box protein family, which binds and can target cyclin E for its ubiquitin-mediated degradation. Mutations of the *FBXW7* gene are reported in ovarian and breast cancer cell lines, as well as in appendiceal cancers [37,209], including *appendiceal adenocarcinomas, mucinous adenocarcinomas*, and *signet ring cell adenocarcinomas of the appendix*, where they occur in 5–10% of cases, and in *well-differentiated neuroendocrine tumors of the appendix* [37].

The *IRX6* gene (16q12.2) encodes the IRX6–*Iroquois Homeobox Protein 6* (446 amino acids; 48,240 Da), whose predicted functions include cell development, neuron differentiation, and regulation of transcription, and retina morphogenesis in the camera-type eye. Its anomalies lead to transient refractive change and aniseikonia [210] since mutations in *the IRX6* gene are found in some cases of *mucinous neoplasms of the appendix* [7].

The *KRT37* gene (17q21.2) encodes KRT37–*Keratin 37* (449 amino acids; 49,747 Da), which is found in hair and nails, and whose defects are identified in multiple types of cataract 1 and *mucinous neoplasms of the appendix* [7,211].

The *MED12* gene (Xq13.1) encodes the MED12–*Mediator Complex Subunit 12* (2177 amino acids; 243,081 Da), part of the Mediator complex, with a mass of 1.2 MDa and which, after binding to the CDK8 subcomplex, pre-initiates transcription. The MED12 protein is essential for CDK8 activation. Defects of the *MED12* gene are present in the X-linked Opitz–Kaveggia and Lujan–Fryns syndromes, and mutations of this gene are reported in appendiceal cancers in general [212] and in a single case of *low-grade appendiceal mucinous neoplasm* [32].

The *MTIF2* gene (2p16.1) encodes the MTIF2–*Mitochondrial Translational Initiation Factor 2* protein (727 amino acids; 81,317 Da), essentially initiating protein synthesis and in the hydrolysis of GTP during the formation of the 70S ribosomal complex. Diseases associated with defects in *the MTIF2* gene are combined oxidative phosphorylation deficiency 39 and combined oxidative phosphorylation deficiency 3 [213], while mutations in its sequence are reported in some cases of *mucinous neoplasms of the appendix* [7].

The *MUC16* gene (19p13.2) encodes the high-molecular-weight, O-glycosylated MUC16–*Mucin 16, Cell Surface-Associated* protein (14,507 amino acids; 1,519,175 Da). As a member of the mucin family, MUC16 forms a mucous barrier over the apical surfaces of the epithelia, protecting them against particles and infectious agents. Defects of the MUC16 gene are found in some diseases, including clear-cell adenocarcinoma and cystadenocarcinoma [214]. Together with MUC17, MUC16 is expressed in pancreato-biliary and small intestinal cancers. In appendiceal cancers, mutations in *the MUC16* sequence are identified in some cases of *mucinous neoplasms of the appendix* [7,32]. Furthermore, MUC16 is expressed in *mucinous adenocarcinomas of the appendix*, showing cytosolic expression and being related to lymph node metastasis since, in appendiceal carcinomas, its expression seems to be lowered [215].

The *OCA2* gene (15q12-q13.1) encodes the OCA2 Melanosomal Transmembrane Protein (838 amino acids; 92,850 Da), probably involved, within melanocytes, in the transport of tyrosine, from which melanin, the pigment in the integument of mammals, is derived. The *OCA2* gene may be involved in determining eye color in humans. Defects in this gene lead to diseases such as oculo-cutaneous albinism type II and variations in skin/hair/eye pigmentation [216]. In appendiceal cancers, some mutations in the *OCA2* gene are reported in *mucinous neoplasms of the appendix* [7], with no known significance in disease progression.

Protocadherin genes *PCDH10* (4q28.3) and *PCDH17* (13q21.1) belong to the protocadherin gene family, included in the cadherin superfamily. *Protocadherin 10* (1040 amino acids; 112,936 Da) and *Protocadherin 17* (1159 amino acids; 126,229 Da) may play roles in specific cell–cell connections in the brain; protocadherin 10 seems to be involved in inhibiting cancer cell motility and cell migration. Defects on these genes are found in some diseases, including developmental and epileptic encephalopathy 9 and Dravet syndrome [217,218], and mutations in both genes are reported in a small number of *mucinous neoplasms of the appendix* cases [7].

The *POM121L12* gene (7p12.1) encodes the POM121L12–*POM121 Transmembrane Nucleoporin Like 12* protein (296 amino acids; 31,848 Da), whose predicted activity is to enable nuclear localization sequence binding activity, and, as a nuclear pore structural constituent, it may aid in RNA export from nucleus and protein import inside it. Among diseases related to POM121L12, defects are phosphoserine phosphatase and serine deficiencies [219]. In addition, the *POM121L12* gene is mutated in a few cases of *mucinous neoplasms of the appendix* [7].

The *PRDM1* gene (6q21) encodes the PRDM1-PR/SET Domain 1 protein (825 amino acids; 91,771 Da), repressing beta-interferon gene expression and stimulating B cell maturation. However, its defects are present in some diseases, including plasmablastic and B-cell lymphomas [220], and the c.711A>T point mutation in the *PRDM1* gene is reported in a single case of *low-grade appendicular mucinous neoplasm* [32].

The *PTCHD3* gene (10p12.1) encodes the PTCHD3–*Patched Domain Containing 3* protein (954 amino acids; 107,689 Da). Probably expressed in the sperm midpiece and active in the membrane, the PTCHD3 protein seems to participate in sperm development or function, with no critical involvement in spermatogenesis and fertility. Defects in the *PTCHD3* gene are present in some diseases, including kidney leiomyosarcoma and multiple cataracts [221]. In appendiceal cancers, mutations in the gene *PTCHD3* are present in *mucinous neoplasms of the appendix* [7] with no known significance.

The *RNF43* gene (17q22) encodes the RNF43–*Ring Finger Protein 43* (783 amino acids; 85,722 Da), which probably negatively regulates the WNT signaling pathway, its defects being identified in sessile serrated polyposis cancer syndrome and colorectal cancer, as well as in multiple tumor types, including colorectal and endometrial cancers [222]. For example, in appendiceal cancers, mutations in *the RNF43* gene are reported in *low-grade appendiceal mucinous neoplasms* [23,30], *high-grade appendiceal mucinous neoplasms*, *appendiceal adenocarcinomas*, and *sessile serrated lesions without dysplasia* [23].

The *SNTG1* gene (8q11.21) encodes the SNTG1–*Syntrophin Gamma 1* protein (517 amino acids; 57,969 Da). As a member of the syntrophin family, the SNTG1 protein mediates dystrophin binding, being involved in gamma-enolase trafficking to the plasma membrane, enhancing its neurotrophic activity and probably organizing the subcellular localization of certain proteins. Some mutations in the *SNTG1* gene are identified in various scoliosis types [223] and, in some cases, in *mucinous neoplasms of the appendix* [7].

The *SOX9* gene (17q24.3) encodes the SOX9–*SRY-Box Transcription Factor 9* (509 amino acids; 56,137 Da), involved in chondrocyte differentiation. Along with steroidogenic factor 1, SOX9 regulates the AMH-anti-Muellerian hormone gene transcription. Deficiencies in *SOX9* gene function are found in some diseases, including campomelic dysplasia and 46, Xy sex reversal 10 [224]. In addition, frameshift mutations of *SOX9* are reported in a few *appendiceal goblet cell adenocarcinomas* [7].

The *STK11* gene (19p13.3) encodes the STK11–*Serine/Threonine Kinase 11* (433 amino acids; 48,636 Da), a tumor suppressor which regulates cell polarity and energy metabolism. Among the disorders associated with STK11 malfunctions are Peutz–Jeghers syndrome and skin, pancreatic, and testicular cancers [225]. In addition, in appendiceal cancers, mutations in the *STK11* gene are observed in *low-grade appendiceal mucinous neoplasms* [30] and *mucinous neoplasms of the appendix* [16,32]. 

The *TSC1* (9q34) and *TSC2* (16p13.3) genes encode two tumor suppressor subunits, TSC1–*Subunit 1* or *Hamartin* (1164 amino acids; 129,767 Da) and TSC2–*Subunit 2* or *Tuberin* (1807 amino acids; 200,608 Da) of the TSC complex, which negatively regulates anabolic cell growth. Disorders associated with *TSC* genes include tuberous sclerosis and lymphangioleiomyomatosis [226,227]. In appendiceal cancers, the TSC1 protein bears a His732Tyr modification in a case of *low-grade appendiceal mucinous neoplasms* [32], and the *TSC2* gene is mutated in appendiceal cancers, with no specification about their type.

## 8. Microsatellite Instability in Appendix Tumors

In addition to genetic mutations identified in all types of appendiceal cancers, there are reports of microsatellite instability (MSI) and small repetitive segments of one, two, or three nucleotides spread in non-coding regions between or within genes. Microsatellite instability (MSI) is a condition caused by the alteration of DNA mismatch repair (MMR) mechanisms, resulting in the accumulation of mutations in nucleotide repeats. These mutations primarily occur in the coding regions of cancer-related genes like TGFβRII, PTEN, and BAX [228].

The DNA mismatch repair (MMR) system plays a crucial role in maintaining the integrity of the genome. It involves several proteins, such as MLH1, MLH3, MSH2, MSH6, MSH3, PMS2, PMS1, and Exo1. MMR proteins work together to identify errors in DNA, which can occur through various mechanisms. The detection of DNA errors is carried out by MSH2/MSH6 and MSH2/MSH3 heterodimers. These heterodimers recognize and bind to mismatches in the DNA sequence. Subsequently, the MLH1/PMS2 complex is recruited to the error site and introduces nicks in the DNA strand, initiating the repair process [229].

Loss of the primary partners (MLH1 and MSH2) results in the loss of the entire heterodimer, while loss of the secondary partners (PMS2 and MSH6) does not lead to the loss of the heterodimer. Mutations or alterations in the MMR proteins can lead to microsatellite instability (MSI), characterized by the accumulation of errors in microsatellite regions of the DNA [230].

Microsatellite instability can arise from different causes. It can be caused by point mutations in *MMR* genes, such as *MLH1* and *MSH2*, which disrupt their normal function. Errors during DNA replication, where the DNA polymerase slips and introduces errors in repetitive DNA sequences, can also contribute to MSI. Additionally, insertions or deletions of bases in microsatellite regions can lead to MSI [231].

Aside from genetic factors, other aberrations can contribute to the MSI phenotype. For example, hypermethylation of the MLH1 promoter can silence *MLH1* gene expression and result in MSI. Epigenetic inactivation of *MSH2* or *MLH1*, downregulation of MMR genes by microRNAs, and slipped strand mating errors (SSMs) are other factors associated with MSI [229].

Microsatellite instability is particularly associated with Lynch syndrome (LS), an inherited genetic disorder caused by germline mutations in *MMR* genes. LS is commonly observed in colorectal and endometrial cancer. However, it can also affect other types of cancer, including ovarian, gastric, hepatobiliary tract, upper urinary tract, pancreatic, brain, and skin cancer [232,233].

Microsatellite instability can manifest in both familial and sporadic colorectal cancer (CRC). Familial cases, comprising approximately 2–3% of all CRC cases [234], are associated with inherited mutations in DNA mismatch repair genes, namely, *MLH1*, *MSH2*, *MSH6*, or *PMS2*. In rare instances, a deletion in the last exon of the *EPCAM* gene can cause *MSH2* methylation, resulting in heritable MSI.

In contrast, sporadic cases of MSI-related CRC are more common and primarily arise from epigenetic silencing of the *MLH1* gene through promoter hypermethylation. This hypermethylation often occurs alongside more generalized methylation of CpG islands, a phenomenon known as the CpG island methylator phenotype (CIMP) [234,235]. Detected in several types of human cancers, most prominently gastrointestinal and endometrial cancers [234,236], MSI is also reported in some appendiceal cancers, such as *appendiceal adenocarcinomas* [237] and *non-mucinous* [234] and *mucinous adenocarcinomas of the appendix* [238]. In cancers, MSI appears to result in a more favorable disease course and prognosis than microsatellite stability. However, the response to 5 fluorouracil (5- FU)-based chemotherapy is poorer [234]. Emile and colleagues performed a retrospective cohort analysis including 1681 patients, 211 of whom were positive for MSI. This study aimed to assess the impact of MSI on the overall survival of patients with appendiceal adenocarcinoma, stratified by disease stage, tumor histology, and patient demographics. They concluded that the MSI status of appendiceal adenocarcinomas did not significantly impact survival, except for stage IV disease, in which a survival benefit of MSI was noted [239].

## 9. Comparison of Genetic Aberrations in Appendiceal, Colorectal, Small Bowel, and Gastric Cancers

**Figure 9:**
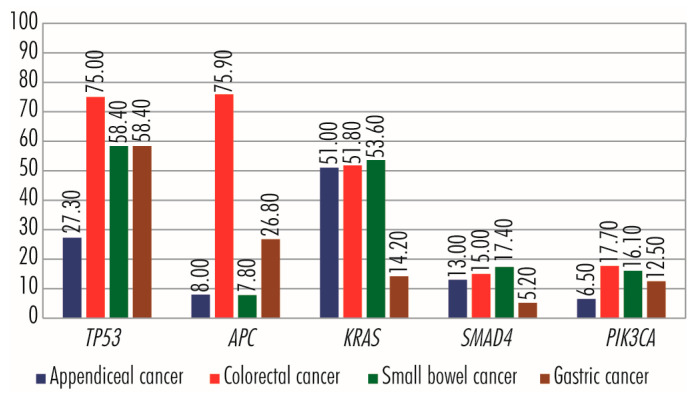
Comparative analysis of gene mutation frequency in appendiceal, colorectal, small bowel, and gastric cancers. According to it, the frequency of mutations in *TP53* and *PIK3CA* genes is lower in appendiceal tumors compared to the other tumor types, and the frequency of mutations in *APC* gene in appendiceal cancers is comparable to that in small bowel tumors, differentiating appendiceal tumors from tumors of the middle and lower digestive tract. However, the frequency of mutations in the *KRAS* and *SMAD4* genes is comparable to the frequency of mutations in these genes in colorectal and small bowel cancers, indicating the possibility of common genetic causes for all three tumors.

Frequently, appendiceal cancers are treated in a similar manner to colorectal cancers, although the former category represents a distinct and unique pathogenetic entity. Thus, mutations in the *TP53* and *APC* genes are present in 27.3% and ~8% of appendiceal cancers, 75% and 75.9% of colorectal cancers, 58.4% and 7.8% of small bowel cancers, and 58.4% and 26.8% of gastric cancers. On the other hand, the frequency of *KRAS* mutations in appendiceal cancers (51%; with a maximum of 56% in adenocarcinomas) is close to that in colorectal cancers (51.8%) and small bowel cancers (53.6%), but much higher compared to gastric cancers (14.2%). The *SMAD4* gene is mutated in about 13% of appendiceal cancers, which is close to that found in colorectal cancers (15%) and small bowel tumors (17.4%), but higher than in gastric cancers (5.2%). *PIK3CA* gene mutations occur with higher frequency in colorectal (17.7%), small bowel (16.1%), and gastric (12.5%) cancers and with lower frequency in appendiceal cancers (~6.5%) [9,13,240] (Figure 9). An interesting feature is the co-occurrence or mutual exclusion of mutations in significant pairs of genes in appendiceal cancers, depending on the time of disease onset. Thus, in cancers that occur early in individuals’ lives (age < 50 years), mutations in *SMAD4* and *PIK3CA* tend to co-occur, whereas mutations in *TP53* and *GNAS* and mutations in *SOX9* and *KRAS* or *TP53* tend to be mutually exclusive. In late-onset cancers (age ≥ 50 years), only mutations in the *GNAS* and *TP53* gene pair are mutually exclusive, while mutations in *GNAS* and *KRAS* or *TP53* genes, in *PIK3CA* and *APC* or *KRAS* genes, in *TP53* and *KRAS* or *SMAD4* genes, and in *ATM* and *SMAD4* genes tend to occur together [13] (Table 1).

## 10. Discussion

Through a review of the literature published up to October 2022, 105 genes have been identified to have mutations in appendiceal cancers (Figure 1).

Of these, 42 genes are included in nine essential signaling pathways in cancer (Figure 2), the rest being more or less involved in tumor progression. Thus, 13 genes, *KRAS*, *HRAS*, *NRAS*, *BRAF*, *EGFR*, *ERBB2*, *ERBB4*, *MET*, *FGFR13*, *KIT*/*cKIT*, *MYC*/*cMYC*, and *PLCG2*, are part of the RAS–RAF–MEK–ERK signaling pathway, which in cancer stimulates proliferation and angiogenesis. Physiologically, genes in this signaling pathway are only/predominantly active during the embryo–fetal period, when they drive tissue growth and differentiation. However, once these processes become much slower in adulthood, these genes are more or less silenced. Due to mutations or epigenetic events, their activation transforms them into oncogenes and contributes to tumor development. This occurs both at transmembrane receptors such as *EGFR*, *ERBB2*, *ERBB4*, *MET*, *FGFR1*–*3*, and *KIT*/*cKIT*, which take up extracellular signals, and at proteins in the cytosol such as *KRAS*, *HRAS*, *NRAS*, *BRAF*, *MYC*/*cMYC*, and *PLCG2*, responsible for their transduction to the nucleus, with *KRAS* and *BRAF* genes undergoing activating mutations in a huge number of appendiceal cancers. Most mutations in genes constituting the RAS–RAF–MEK–ERK signaling pathway are reported in *mucinous adenocarcinomas of the appendix* (nine genes), followed by *appendiceal goblet cell adenocarcinomas* and *well-differentiated neuroendocrine tumors of the appendix*, with six genes each. None of the genes in this pathway appear mutated in *mucinous neoplasms of the appendix*, *appendiceal adenocarcinomas*, and *signet ring cell adenocarcinoma*.

Frequently, mutations in the *KRAS* gene occur concomitantly with mutations in the *GNAS* gene in the RAP1 signaling pathway, which involves numerous biological processes, including invasiveness, adhesion, cell migration and polarization, and tumor metastasis. Frequent mutations in appendiceal cancers are also reported in the *TP53* gene, this time being inactivating. The *TP53* gene helps determine the fate of cells whose genetic material is damaged, and its inactivation leads to cells remaining on the path of tumor transformation. 

Another pathway mutated in appendiceal cancers is PI3K–PKB/AKT, in which eight genes, *AKT1*, *ITGA11*, *PIK3C2B*, *PIK3CA*, *PTEN*, *CDKN1B*, *CDKN2A*, and *RB1*, in addition to those affecting receptors typical to the RAS–RAF–MEK–ERK signaling pathway, are mutated. The eight genes encode cytosolic proteins, which transduce signals from transmembrane receptors to the nucleus through the range of molecules they activate. They are involved in the most critical tumor processes, proliferation, evading apoptosis, and angiogenesis. Among appendiceal cancers, some *low-grade appendiceal mucinous neoplasms* carry mutations in five genes, and *appendiceal goblet cell adenocarcinomas* carry mutations in four genes. 

In the JAK–STAT signaling pathway, in addition to molecules that function as transmembrane receptors, including the RAS–RAF–MEK–ERK and PI3K–PKB/AKT signaling pathways, the *JAK3* gene, its central node, is also mutated in appendiceal cancers, and through which signals necessary for both evasions of apoptosis and support of angiogenesis are transmitted. 

Resistant tumors are known to develop alternative pathways for survival so that if one of these is inactivated, the others will take its place. In some appendiceal cancers, the alternative to the PI3K–PKB/AKT signaling pathway is the activation of the WNT pathway, which also signals cell proliferation and evasion of apoptosis. Of the members of this signaling pathway, the *APC*, *AXIN1*, *TCF7L2*, and *RHOA* genes are most frequently mutated, the latter encoding a low-molecular-mass protein that contributes to cell invasiveness, adhesion, migration, and polarization, and tumor metastasis. This function overlaps with the more frequently mutated *GNA11* and *GNAS* genes of the RAP1 signaling pathway. Members of the WNT signaling pathway are more frequently mutated in *low-grade appendiceal mucinous neoplasms* and *appendiceal goblet cell adenocarcinomas*.

Tumor growth requires an increased supply of oxygen and nutrients, the lack of which activates the HIF1–VEGF signaling pathway to neo-form blood vessels. In tumors, these have an altered structure, with other types of cells, including tumor cells, coexisting in their walls alongside normal endothelial cells, or lacunae, which allow the transformed cells to enter and metastasize elsewhere in the body. In appendiceal cancers activating mutations in the HIF1–VEGF signaling pathway are reported in three genes, *EP300*, *CREBBP*, and *VEGFR2*. In addition to the classical HIF1–VEGF signaling pathway, tumor angiogenesis is stimulated by two alternative mechanisms. One is through the NOTCH pathway. The *NOTCH1*, *NOTCH3*, *NOTCH4*, and *FLT1/VEGFR1* genes are mutated/reactivated in appendiceal cancers, and the other is through inactivation of the tumor suppressor genes *SMAD2–4* and *TGFBR1–2* in the TGFβ signaling pathway. While in the case of members of the NOTCH signaling pathway, mutations are rare and not prevalent in specific tumor types, in the case of genes in the TGFβ signaling pathway, they are mutated in some cases of *mucinous neoplasms of the appendix* and *low-grade appendiceal mucinous neoplasms*. 

The other genes, which are not included in the nine major cancer signaling pathways, are sporadically mutated, although those in the *RNF43* sequence occur more frequently. Most mutations in these genes are present in *mucinous neoplasms of the appendix*, the generic name for mucinous tumors, and of these, in *low-grade appendiceal mucinous neoplasms*. 

**Figure 10:**
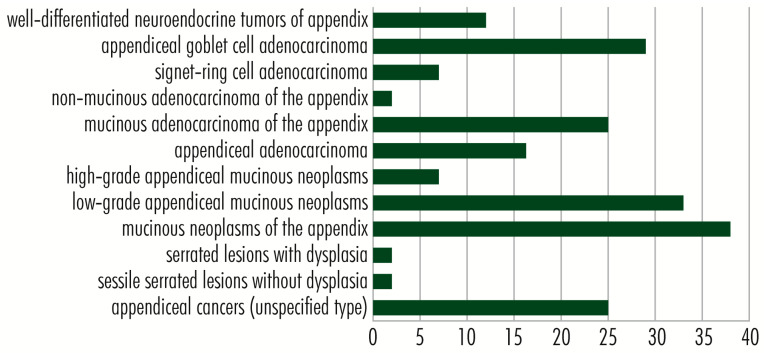
Number of genes in which mutations occur in appendiceal cancers.

In the analysis of the total number of mutated genes in various types of appendiceal cancers, *mucinous neoplasms of the appendix* are ranked first (Figure 10), with mutations reported in 38 genes out of 105, i.e., *ITGA11*, *TGFBR1*, *TGFBR2*, *SMAD2*, *SMAD3*, *SMAD4*, *ABCA7*, *ANKRD24*, *APOB*, *CNTNAP2*, *COL5A3*, *COL6A3*, *CRY2*, *CTNNB1*, *DCLK1*, *DOCK3*, *DOK6*, *EEF1A1*, *EPHA10*, *FAT4*, *IDH2*, *IRX6*, *KRT37*, *LAMA1*, *MTIF2*, *MUC16*, *OCA2*, *PCDH10*, *PCDH17*, *POM121L12*, *PRKACA*, *PTCHD3*, *PTPN11*, *SNTG1*, *SPTA1*, *STK11*, *TRPS1*, and *ZNF469*, of which *low-grade appendiceal mucinous neoplasms* carry mutations in 33 genes: *KRAS*, *BRAF*, *MET*, *TP53*, *AKT1*, *PIK3C2B*, *PIK3CA*, *CDKN2A*, *RB1*, *JAK3*, *APC*, *AXIN1*, *GNAS*, *CREBBP*, *NOTCH1*, *NOTCH4*, *TGFBR2*, *SMAD2*, *SMAD4*, *ARID1A*, *ATM*, *CTNNB1*, *DIS3*, *FANCA*, *FAT4*, *FH*, *MED12*, *PRDM1*, *RAD51C*, *RNF43*, *SMARCA4*, *STK11*, and *TSC1*. 

**Figure 11:**
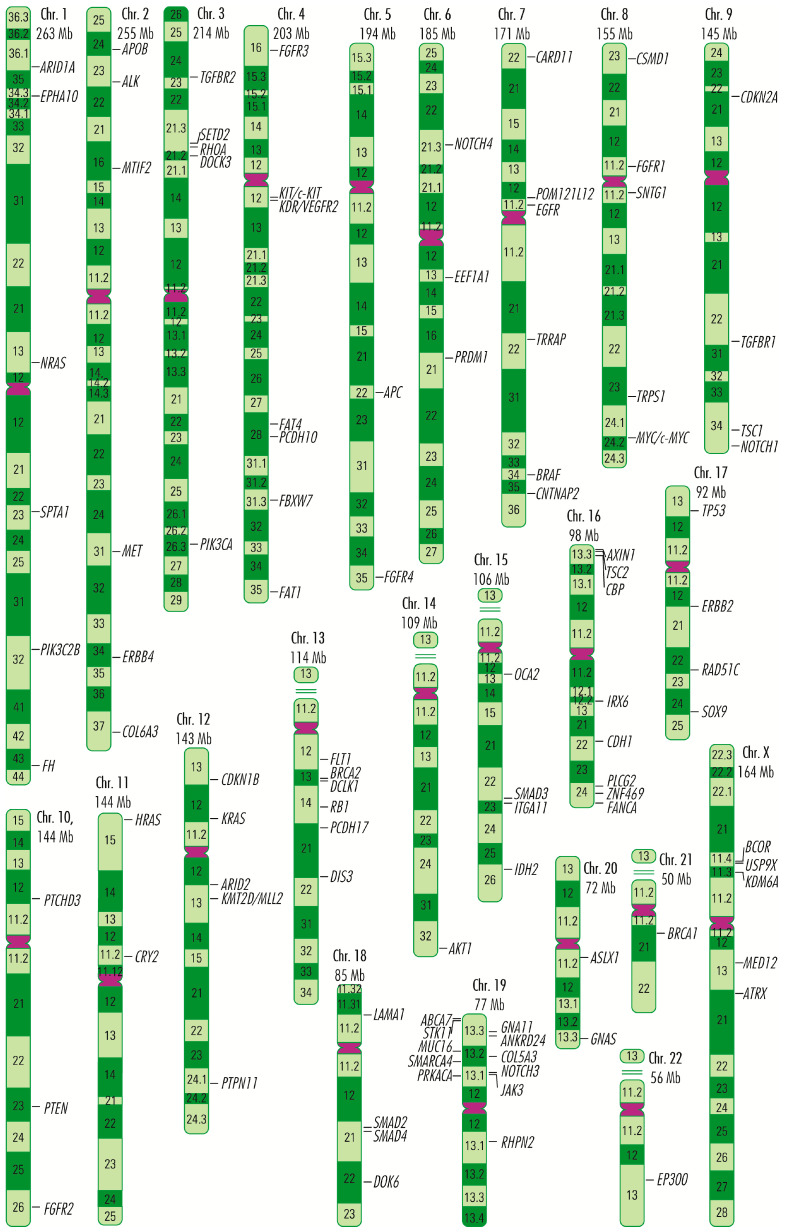
Chromosome distribution of genes that are mutated in appendiceal cancers.

Next are appendiceal goblet cell adenocarcinomas, in which 29 mutated genes, *KRAS*, *HRAS*, *NRAS*, *BRAF*, *ERBB2*, *MYC/cMYC*, *TP53*, *PTEN*, *CDKN1B*, *CDKN2A*, *RB1*, *APC*, *RHOA*, *GNAS*, *NOTCH1*, *TGFBR2*, *SMAD4*, *ARID1A*, *ARID2*, *ATM*, *CDH1*, *CTNNA1*, *CTNNB1*, *KDM6A*, *KMT2D*, *RHPN2*, *SOX9*, *TRRAP*, and *USP9X*, and mucinous adenocarcinomas of the appendix, with 25 genes, *KRAS*, *HRAS*, *NRAS*, *BRAF*, *ERBB2*, *FGFR1*, *FGFR2*, *FGFR3*, *KIT/cKIT*, *MYC/cMYC*, *TP53*, *PIK3CA*, *RB1*, *JAK3*, *APC*, *GNA11*, *GNAS*, *KDR/VEGFR2*, *TGFBR2*, *ARID1A*, *ATM*, *BRCA1*, *BRCA2*, *CDH1*, *FBXW7*, and *MUC16*, in which mutations are identified. In the other types of appendiceal tumors, the number of genes in which mutations are present is lower. Among the loci that concentrate the most genes in which mutations occur in appendiceal cancers, 19p13 ranks first, with ten genes; other loci such as 3p21.2-3p21.3, 16q24, 16p13.3, and Xp11.3-11.4 each concentrate three genes (Figure 11). These loci could be used for searching possible genetic markers for appendiceal cancers.

Chromosome 18q is the location of the *DCC*, *DPC4* (*SMAD4*; *MADH4*), and *JV-18* (*SMAD2*; *MADH2*) genes, and loss is frequent in colorectal adenocarcinoma [241]. The status of chromosome 18q has also been shown in previous studies to be an independent prognostic factor in patients with stage II and III colon carcinomas, and tumors without loss are associated with better clinical outcomes. DPC4 is a tumor suppressor gene with frequent alterations in pancreatic adenocarcinomas [242]. Maru et al. studied the genetic alterations, including loss of chromosome 18q (location of *DCC*, *DPC4*, and *JV-18* genes) and mutations of the *DPC4* (*SMAD4*) and beta-catenin genes in 28 appendiceal adenocarcinomas. Chromosome 18q loss was present in 57% of appendiceal carcinomas. Mutation of the *DPC4* gene was present in 14% (three of 22) of the carcinomas occurring in one tumor with chromosome 18q loss and in two with unassessed chromosome 18q status. The authors concluded that the presence of chromosome 18q loss and *DPC4* mutations in appendiceal adenocarcinomas suggests the involvement of *DPC4* and nearby genes on chromosome 18q (*DCC* and/or *JV-18*) in the pathogenesis of appendiceal adenocarcinomas [243]. Davison et al. performed a study including 109 disseminated appendiceal mucinous neoplasms aiming to correlate *SMAD4* immunohistochemical expression with tumor grade and assess the prognostic significance of *SMAD4* expression in predicting overall survival. Their results indicate that loss of *SMAD4* immunohistochemical expression is associated with loss of heterozygosity at chromosome 18q and is always associated with aggressive histologic features in disseminated appendiceal mucinous neoplasms. *SMAD4* immunohistochemistry may be a practical instrument in select cases of disseminated appendiceal neoplasia, in which the distinction between low-grade and high-grade tumors is difficult [241].

## 11. Future Perspectives

The appendix tumor microenvironment consists of cellular and noncellular components: the former includes the immunocompetent cells, while the latter represents the supporting stroma. Particularly in carcinoids, the immune cell reaction can be explicated by tumor-infiltrating lymphocytes, which, in some circumstances, may arrange around and inside the tumor briskly, influencing the prognosis favorably. This active reaction has to be distinguished from any preexisting inflammatory condition of the appendix and superimposed tumor complications, such as infection or ischemia. In practice, the appendix TME is a complex framework with immunological, mechanic, and metabolic functions, all supported by marked neo-lymphoangiogenesis [244].

Current surgical strategies for localized well-differentiated appendiceal cancers may vary from simple appendicectomy to right hemicolectomy, depending on tumor size and invasion patterns. Because the diagnosis is usually established during the histological examination of an appendectomy specimen, it is essential to identify the subgroup of patients who will require further therapy. Recently, significant advances have been made in the management of appendix cancers, and novel therapeutic options are available for patients with metastatic disease; cytotoxic chemotherapy should be used only for patients with high-grade tumor burden who have no other therapeutic options. Although poorly differentiated appendiceal cancers are rare, their management involves platinum etoposide chemotherapy. Very few trials have included patients with these tumors; the data are scant. Therefore, it is crucial to evaluate many factors, including surgical respectability even in the metastatic setting, the status of somatostatin avidity, histological grade, and tumor burden, to select the appropriate personalized treatment for every patient through multidisciplinary discussion by experts [245]. Surgical intervention is determined in mucinous neoplasm according to the mesoappendix’s pathological subtype and involvement. Regarding the need for additional surgical intervention or medical treatment for patients with tumors, histopathological results must be followed carefully after appendectomy.

The review of these scattered cases raises a question regarding the true incidence of appendiceal involvement in gastric tumors, particularly the asymptomatic metastases, the answer to which leads to re-questioning the benefit–risk ratio of performing appendectomy for every gastric oncology surgery. Given reported patients’ prognoses, guidelines on managing such cases are also needed. Clinical and morphological analyses of cases from the practice of malignant tumors of the appendix (neuroendocrine tumor and adenocarcinoma) will be valuable and exciting for the medical community and should stimulate cancer vigilance in physicians [246].

Traditional cancer models should be generated for rare cancers, even using conventional methods, because the urgent need for rare cancer models has long been neglected. We need targeted and conscious efforts to generate “orphan models” to promote rare cancer research, as we currently have for orphan drugs [247]. Such model creation should be conducted in a non-competitive way. In one example, patient-derived rare cancer models are being generated at the National Cancer Center Research Institute, Tokyo, Japan, in global collaboration with multiple institutions. These models are then characterized and applied to translational research by our group and, in parallel, are delivered to multiple research groups.

Understanding that the paucity of cancer models is the fundamental problem in rare cancer research is essential. It is necessary to intensively promote the development of rare cancer models and to share them freely within the research community. This requires multi-institutional studies, as clinical samples are rare, and the research that single research groups can perform is limited. Such efforts and the models they produce will enable future generations to better understand cancer biology and develop novel biotechnologies and treatments, dramatically improving rare cancer outcomes. By creating novel patient-derived cancer models, we can change the future of rare cancer research.

## 12. Conclusions

An analysis of the presence of mutations of 105 genes in appendiceal cancers through the lens of the literature reviewed supports the view that in most of them, the inactivation of tumor suppressor genes, such as *TP53* and *SMAD4*, is required in parallel with the reactivation of genes with oncogenic potentials, such as *KRAS*, *GNAS*, and *BRAF*, which support the main tumor processes, cell proliferation, angiogenesis, and evasion of apoptosis. Frequently, genes belonging to the RAS–RAF–MEK–ERK signaling pathway are mutated as the primary mechanism of appendix tumorigenesis, activating alternative pathways such as PI3K–PKB/AKT, NOTCH, RAP1, TGFB, and WNT. 

Of all appendiceal cancers, the most mutated genes are reported in mucinous neoplasms of the appendix, not including those in the *RAS–RAF–MEK–ERK signaling pathway*, followed by low-grade appendiceal mucinous neoplasms, appendiceal goblet cell adenocarcinomas, and mucinous adenocarcinomas of the appendix, in which this signaling pathway is most frequently affected, showing its importance in their tumorigenesis. Microsatellite instability rarely occurs in appendix cancers, reported only in adenocarcinomas.

## Figures and Tables

**Table 1:** Significant pairs of genes whose mutations co-occur or are mutually exclusive in early and late-onset appendiceal cancers.

First Mutated Gene	Second Mutated Gene	Relation
Early-onset appendiceal cancers		
P53	GNAS	Mutual exclusivity
SOX9	KRAS/TP53	Mutual exclusivity
SMAD4	PIK3CA	Co-occurrence
Late-onset appendiceal cancers		
GNAS	KRAS	Co-occurrence
PI3KCA	APC/KRAS	Co-occurrence
TP53	KRAS	Co-occurrence
GNAS	TP53	Mutual exclusivity
SMAD4	TP53/ATM	Co-occurrence

## Data Availability

All data can be found in the text.
